# Glycolytic regulation of cell rearrangement in angiogenesis

**DOI:** 10.1038/ncomms12240

**Published:** 2016-07-20

**Authors:** Bert Cruys, Brian W. Wong, Anna Kuchnio, Dries Verdegem, Anna Rita Cantelmo, Lena-Christin Conradi, Saar Vandekeere, Ann Bouché, Ivo Cornelissen, Stefan Vinckier, Roeland M. H. Merks, Elisabetta Dejana, Holger Gerhardt, Mieke Dewerchin, Katie Bentley, Peter Carmeliet

**Affiliations:** 1 Department of Oncology, Laboratory of Angiogenesis and Vascular Metabolism, KU Leuven, Herestraat 49 box 912, B-3000 Leuven, Belgium; 2 Vesalius Research Center, Laboratory of Angiogenesis and Vascular Metabolism, VIB, Herestraat 49 box 912, B-3000 Leuven, Belgium; 3 Life Sciences Group, Centrum Wiskunde and Informatica, Science Park 123, 1098 XG Amsterdam, The Netherlands; 4 Mathematical Institute, Leiden University, Niels Bohrweg 1, 2333 CA Leiden, The Netherlands; 5 Department of Immunology, Genetics and Pathology, Rudbeck Laboratory, Uppsala University, 751 85 Uppsala, Sweden; 6 FIRC Institute of Molecular Oncology, Via Adamello 16, 20139 Milan, Italy; 7 Department of Oncology and Hemato-Oncology, Milan University, 20139 Milan, Italy; 8 Department of Oncology, Vascular Patterning Laboratory, KU Leuven, Herestraat 49 box 912, B-3000 Leuven, Belgium; 9 Vesalius Research Center, Vascular Patterning Laboratory, VIB, Herestraat 49 box 912, B-3000 Leuven, Belgium; 10 Integrative Vascular Biology Laboratory, Max-Delbrück-Center for Molecular Medicine, Robert-Rössle-Strasse 10, D-13125 Berlin, Germany; 11 Department of Pathology, Computational Biology Laboratory, Beth Israel Deaconess Medical Center, Harvard Medical School, 330 Brookline Avenue, Boston, MA 02215, USA

## Abstract

During vessel sprouting, endothelial cells (ECs) dynamically rearrange positions in the sprout to compete for the tip position. We recently identified a key role for the glycolytic activator PFKFB3 in vessel sprouting by regulating cytoskeleton remodelling, migration and tip cell competitiveness. It is, however, unknown how glycolysis regulates EC rearrangement during vessel sprouting. Here we report that computational simulations, validated by experimentation, predict that glycolytic production of ATP drives EC rearrangement by promoting filopodia formation and reducing intercellular adhesion. Notably, the simulations correctly predicted that blocking PFKFB3 normalizes the disturbed EC rearrangement in high VEGF conditions, as occurs during pathological angiogenesis. This interdisciplinary study integrates EC metabolism in vessel sprouting, yielding mechanistic insight in the control of vessel sprouting by glycolysis, and suggesting anti-glycolytic therapy for vessel normalization in cancer and non-malignant diseases.

## Abstract (web summary)

Glycolytic regulator PFKFB3 is a key player in vessel sprouting. Here the authors develop a computational model predicting that PFKFB3 drives endothelial cell rearrangement during vessel sprouting by promoting filopodia formation and reducing intercellular adhesion, and empirically validate this prediction.

## Graphical abstract

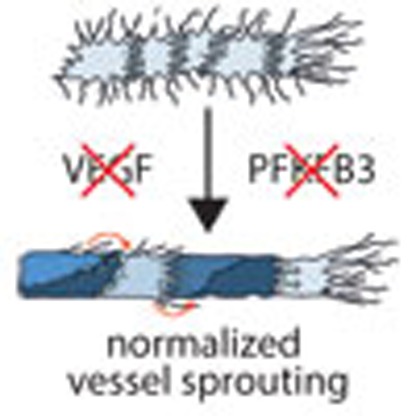

During angiogenesis, a blood vessel sprout is guided by a migrating ‘tip' cell and elongated by proliferating ‘stalk' cells. Lateral DLL4/Notch signalling underlies tip cell selection and regulates the response of endothelial cells (ECs) to the pro-angiogenic signal vascular endothelial growth factor (VEGF). Indeed, by inducing VEGF receptor 2 (VEGFR2) signalling, VEGF activates the EC expressing the highest levels of this receptor. However, VEGFR2 signalling also upregulates DLL4 expression, which activates the Notch1 receptor on neighbouring cells. This, in turn, lowers VEGFR2 expression, thereby rendering these cells less responsive to VEGF, as such creating a ‘salt & pepper' (S&P) pattern of activated and inhibited ECs^1^,^2^. Here we use ‘tip' and ‘stalk' to refer to a cell's relative position in the sprout, and ‘active/activated' and ‘inhibited' to indicate the cellular state. These states are dynamically interchangeable, allowing ECs in a sprout to overtake each other (termed EC rearrangement), thereby ensuring that the most competitive EC leads the sprout^3^,^4^.

Glycolysis promotes EC competitiveness for the tip position^5^. ECs that are instructed to become a stalk cell can still overtake their wild-type (WT) neighbours and become a tip cell through the overexpression of the glycolytic regulator 6-phosphofructo-2-kinase/fructose-2,6-bisphosphatase 3 (PFKFB3)^5^. Genetic and pharmacological inhibition of PFKFB3 reduces sprouting^5^,^6^ and the capacity of ECs to reach the sprout tip^5^, while PFKFB3 overexpression induces opposite effects^5^,^7^. Furthermore, PFKFB3 knockdown (PFKFB3^KD^) in ECs reduces filopodia and lamellipodia formation^5^. Finally, blockade of glycolysis inhibits pathological angiogenesis^6^,^7^,^8^.

EC rearrangement depends on differential VE-cadherin-dependent intercellular adhesion and differential formation of polarized junctional cortex protrusions (referred to as ‘cortical protrusions'). These processes drive EC intercalation and depend on VEGF-Notch signalling^9^. While VEGF promotes VE-cadherin endocytosis^10^, EC motility^2^, forward–rear cell polarity^3^, weak intercellular adhesion and serrated junctions^9^, Notch signalling impairs EC rearrangement^4^ by rendering cells more adhesive and by suppressing cortical protrusions, resulting in ‘straighter' junctions^9^. EC shuffling thus requires actin remodelling^11^,^12^, which is highly ATP consuming^13^ as it can require up to 50% of cellular ATP levels^14^,^15^. In ECs, glycolytic production of ATP is essential for the formation of cytoskeletal protrusions^5^ and the stability of intercellular junctions^16^. In addition, endocytosis of cadherins, which determines the available cadherin levels at the plasma membrane and hence also adhesion, relies on ATP in epithelial cells^17^,^18^,^19^,^20^. However, it remains unknown how glycolysis regulates EC rearrangements during vessel sprouting and, in particular, whether PFKFB3-driven glycolytic production of ATP controls filopodia extension, intercellular adhesion (via an effect on VE-cadherin endocytosis), and formation of cortical protrusions during EC rearrangement.

Here we investigate the link between EC rearrangements and metabolism. By combining computational modelling with experimentation, we identify mechanistic insights into how PFKFB3-driven glycolysis steers EC rearrangements during vessel sprouting, and show that targeting glycolysis in ECs normalizes cell rearrangement and vessel disorganization in disease, meriting further attention for therapy. Throughout this study, we follow an integrated ‘symbiotic' approach^21^, iteratively using *in vitro* and *in vivo* experiments to validate and refine our computational model and to confirm *in silico* predictions.

## Results

### MemAgent-Spring computational model characteristics

**Figure 1:**
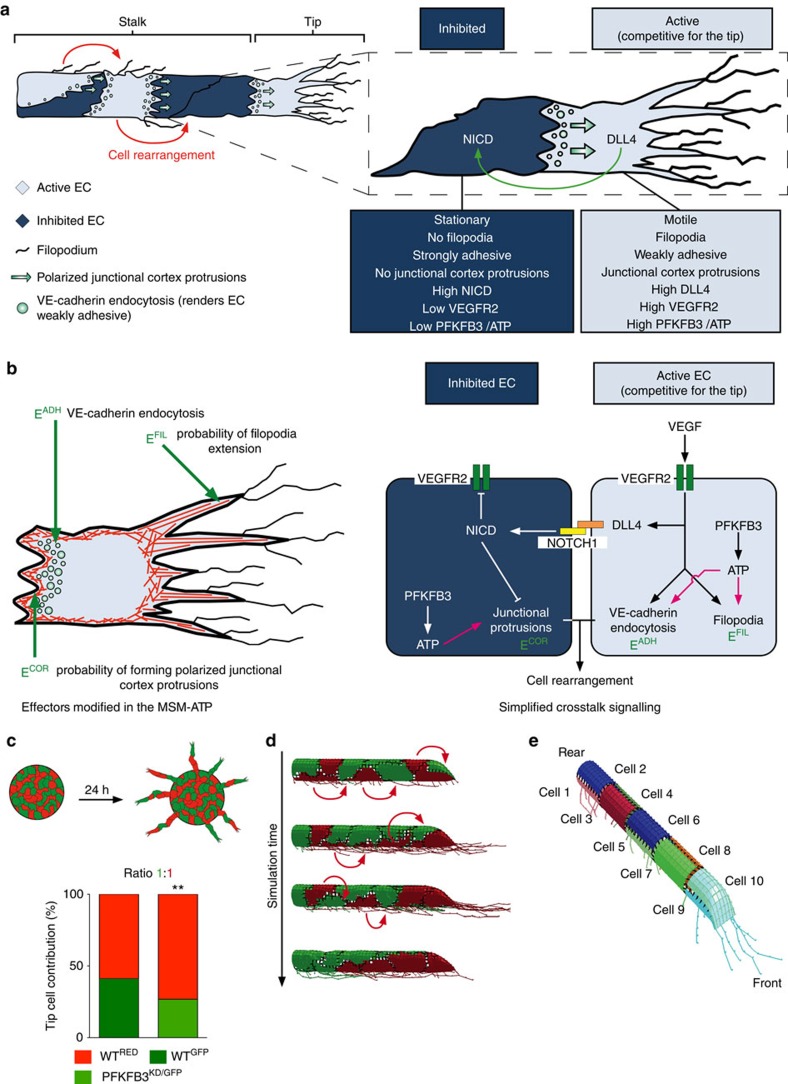
General concept of the study. (**a**) Schematic of a sprout showing the differential properties of its ECs, which are activated or inhibited. Active ECs inhibit adjacent cells through Dll4/Notch signalling, are migratory and competitive for the tip. They overtake other ECs (cell rearrangement) due to higher VE-cadherin endocytosis rates (which makes them weakly adhesive) and formation of junctional cortical protrusions. (**b**) Scheme depicting the effectors modified in the memAgent-Spring ATP model (MSM-ATP) and the signalling pathways included in the MSM-ATP. E^FIL^ (representing filopodial F-actin), E^COR^ (referring to cortical actin), and E^ADH^ (denoting intercellular adhesion) determine respectively, the probability of filopodia extension, the formation of polarized junctional protrusions and cellular adhesion levels, determined by VE-cadherin endocytosis. The pink lines indicate the ATP effector links that were investigated. (**c**) Schematic drawing of the *in vitro* EC spheroid assay. Cells with different genotypes (and corresponding colours) are cultured in a sphere and allowed to sprout for 24 hours upon growth factor stimulation. Subsequently, the colour of the cell at the tip is counted for each sprout, allowing to quantify the tip cell contribution as shown in the graph for 1:1 WT^GFP^:WT^RED^ and PFKFB3^KD/GFP^:WT^RED^ mosaic spheroids. Data are mean±s.e.m.; *n*=30 from 3 donors; **P<0.01; Fisher's exact test; adapted from ref. 5 with permission from Elsevier. (**d**) Screenshots of MSM-ATP simulations, showing how cell positions change over time and how, similar as in the *in vitro* assay, the genotype (colour) of the cell at the end of the *in silico* experiments can be assessed. The red arrows represent the movement of the cell to its next position in the next simulation screenshot. (**e**) Scheme illustrating how MSM-ATP simulations are performed using vessel sprouts consisting of ten ECs. Each colour represents a different EC. ATP, adenosine triphosphate; DLL4, delta-like 4; NICD, Notch1 intracellular domain; PFKFB3, 6-phosphofructo-2-kinase/fructose-2,6-bisphosphatase 3; VE-cadherin, vascular endothelial cadherin; VEGF, vascular endothelial growth factor; VEGFR2, VEGF receptor 2.

For reasons of clarity, we first introduce some key features of the memAgent-Spring computational model (MSM) before describing our new extensions to the model (for full details see Supplementary Note). The MSM is a spatiotemporal, agent-based model in which an *in silico* vessel sprout contains ECs, whose membranes consist of many small computational agents (‘memAgents') that can move on an interlinked surface mesh. The memAgents are connected in the mesh by springs, representing the actin cortex underneath the cell membrane^9^. The MSM recapitulates VEGF/Notch-dependent tip/stalk cell selection as follows: ECs sensing higher levels of VEGF have more active VEGFR2 signalling. These ‘activated' cells express higher DLL4 levels, which activate Notch receptors and thereby ‘laterally inhibit' VEGFR2 signalling in adjacent cells (Fig. 1a,b)^22^. VEGFR2 signalling also increases local actin polymerization, thereby promoting the formation of dynamic filopodia. Since filopodia express VEGFR2 (ref. 23), a positive feedback is generated, whereby VEGFR2 stimulates filopodia to move further into the VEGF gradient, amplifying its own signalling and subsequent DLL4 presentation to neighbouring ECs^24^. Hence, filopodia act as amplifiers of DLL4/Notch-mediated lateral inhibition in the MSM, a form of active perception to probe the environment and arrive at a selection of activated/inhibited states^22^,^25^,^26^. Recently, we incorporated a modified implementation of the Cellular Potts model^27^ into the MSM to allow differential adhesion and cortical protrusion formation, which together drive EC rearrangement^9^.

### The MSM-ATP model

Tip cells are more glycolytic than stalk cells, as they express higher levels of PFKFB3 (ref. 5) (Fig. 1a). Indeed, when PFKFB3^KD^ and WT ECs are mixed in a 1:1 ratio in mosaic EC spheroids, PFKFB3^KD^ ECs contribute to only 22.4% of the tip cells (instead of the expected 50% if they would be as competitive as WT cells)^5^ (Fig. 1c). Likewise, when PFKFB3^KD^ and WT ECs are mixed in a 9:1 ratio, PFKFB3^KD^ cells contribute to only 66.8% of the tip cells (instead of the expected 90%)^5^. We therefore modelled another level of regulation of vessel sprouting as a new extension to the MSM, namely glycolytic ATP production, in order to assess how these ATP levels regulate EC rearrangement. We upgraded the latest MSM version to the MSM-ATP by making three model effectors driving EC migration (filopodia formation, cortical protrusions, intercellular adhesion) dependent on glycolytic ATP levels. We therefore included the following modifier constants: (i) *k*_*FIL*_ to modify the filopodia effector (E^FIL^), reflecting the *in silico* probability of filopodia extension; (ii) *k*_*COR*_ to alter the cortex effector (E^COR^), reflecting the *in silico* probability of forming cortical protrusions that facilitate ECs to move relative to each other; and (iii) *k*_*ADH*_ to change the intercellular adhesion effector (E^ADH^) (Fig. 1b; Methods section and Supplementary Note). Intercellular adhesion is determined by the amount of VE-cadherin on the cell surface, which itself depends on VE-cadherin endocytosis, a process that is stimulated by VEGFR2 signalling^10^. Thus, an EC with high VEGFR2 activity (VEGFR2 phosphorylation) has more VE-cadherin endocytosis, which reduces its adhesion and thereby promotes EC motility. Therefore, E^ADH^ classifies cells as either strongly or weakly adhesive based on the cell's VEGFR2 activity.

We also considered other MSM-ATP effectors that might depend on glycolytic ATP, such as the amount of actin that ECs can accumulate, the stability of filopodia, and the phosphorylation and glycosylation status of VEGFR2. However, since PFKFB3 inhibition did not change VEGFR2 phosphorylation^5^ or VEGFR2 glycosylation^6^, and changing filopodia stability or the amount of actin did not match all features assessed during the elimination process (Supplementary Fig. 1), we did not further consider these processes.

### Simulating EC rearrangement in mosaic vessel sprouts

Our overall objective was to explore whether the competitive disadvantage of PFKFB3^KD^ ECs could be explained by an effect of reduced glycolytic ATP production on filopodia formation and/or EC rearrangement. To identify via which ATP-dependent effector mechanism(s) PFKFB3-driven glycolysis regulates tip cell competition, we varied the aforementioned MSM-ATP effectors, alone or in combination, to simulate the PFKFB3^KD^ phenotype *in silico* (referred to as ‘isPFKFB3^KD^'). Single mechanisms (in which only one effector was modified) included E^FIL^, E^COR^ and E^ADH^, while combinatorial mechanisms enclosed E^FIL/COR^, E^FIL/ADH^, E^COR/ADH^ and E^ALL^. We refer to isPFKFB3^KD^ cells simulated by these respective mechanisms as follows: isPFKFB3^KD-FIL^, isPFKFB3^KD-COR^, isPFKFB3^KD-ADH^, isPFKFB3^KD-FIL/COR^, isPFKFB3^KD-FIL/ADH^, isPFKFB3^KD-COR/ADH^ and isPFKFB3^KD-ALL^.

As depicted in the study outline (Supplementary Fig. 1), we first calibrated the single-effector mechanisms by varying the *k*_*FIL*_, *k*_*COR*_ and *k*_*ADH*_ constants over wide ranges in isPFKFB3^KD^ cells and assessed how these competed in mosaic sprouts with random placement of *in silico* WT (isWT) and isPFKFB3^KD^ ECs using an established approach^9^ (Fig. 1d). We will refer to mosaic sprouts consisting of isPFKFB3^KD^ and isWT ECs, mixed in a 1:1 and 9:1 ratio, as 1:1 or 9:1 isPFKFB3^KD^:isWT sprouts, respectively. Simulations were performed with sprouts containing 10 ECs, with 2 cells per cross-section (Fig. 1e, Supplementary Movie 1). To match the simulations with the *in vitro* results, we developed a computational method to quantify tip cell competition *in silico*, similar to how tip cell competition is measured manually *in vitro* (see Methods section).

We then tested the potential of these mechanisms to explain the PFKFB3^KD^ phenotype by comparing them to published experimental data, and eliminated those that did not match (Supplementary Fig. 1). This was done to identify the mechanism(s) that explained best the role of glycolytic ATP in EC rearrangement and filopodia formation and to ensure the model's predictive capacity. Thereafter, we predicted EC behaviour in experimentally untested conditions, such as Notch inhibition and pathological VEGF levels, and subsequently experimentally validated these *in silico* predictions to ultimately identify the most representative isPFKFB3^KD^ mechanism (Supplementary Fig. 1).

### Regulation of tip cell competition by MSM-ATP effectors

**Figure 2:**
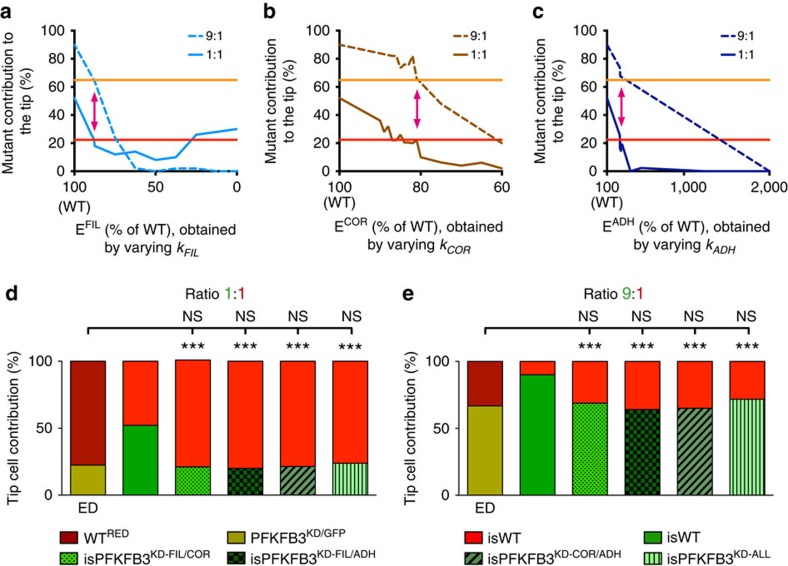
Effector mechanism simulations of tip cell contribution. (**a–c**) Sensitivity analyses of respectively E^FIL^ (**a**), E^COR^ (**b**) and E^ADH^ (**c**) as single mechanism governing tip cell competition, by varying over a wide range values of respectively *k*_*FIL*_, *k*_*COR*_ and *k*_*ADH*_. The results for both the 1:1 (full line) and 9:1 (dashed line) *in silico* PFKFB3^KD^:WT (isPFKFB3^KD^:isWT) mosaic sprouts are shown. The red and orange horizontal line depict the tip cell contribution of PFKFB3^KD^ cells, as obtained experimentally in respectively the 1:1 and 9:1 PFKFB3^KD^:WT sprouts^5^. The combined 1:1 and 9:1 simulation results yielded one specific *k* value (see pink double-headed arrow) for every single effector mechanism. (**d,e**) Combinatorial E^FIL/COR^, E^FIL/ADH^, E^COR/ADH^ and E^ALL^ simulations of tip cell contribution in 1:1 (**d**) or 9:1 (**e**) isPFKFB3^KD^:isWT mosaic sprouts, illustrating that for every combinatorial effector, *k* values for its contributing effectors could be obtained matching the competitive disadvantage for PFKFB3^KD^ cells as observed in the experimental PFKFB3^KD^:WT EC spheroid competition data (experimental data (ED), *n*=30 spheroids from 3 donors). For each combinatorial effector, the same *k* values matched both the 1:1 (first bar in **d**) and 9:1 (first bar in **e**) experimental mosaic PFKFB3^KD^:WT sprout data. The second bar in panel d and e shows the expected 50% (**d**) or 90% (**e**) contribution of isWT cells in 1:1 (**d**) or 9:1 (**e**) isWT:isWT sprouts. *n*=150; ***P<0.001, versus isWT:isWT. ‘NS' (not significant) versus ED; Fisher's exact test.

When modifying each effector mechanism alone in 1:1 isPFKFB3^KD^:isWT sprouts, we identified *k* values for each effector that yielded the exact same tip cell contribution as observed experimentally (Fig. 2a-c, full lines). In fact, for isPFKFB3^KD-FIL^ and isPFKFB3^KD-COR^ ECs, multiple *k* values matched the experimental data. Since we could not select a single *k* value, optimally fitting the experimental data, we used an additional criterion that the *k* values also had to match 9:1 PFKFB3^KD^:WT EC spheroid data. Also for this ratio, we found *k* values that matched the experimental data (Fig. 2a–c, dotted lines). When combining the 1:1 and 9:1 isPFKFB3^KD^:isWT results, we identified a *k* value for each single effector mechanism that optimally matched the experimental results (Fig. 2a–c; Supplementary Note Table 1).

Since it is likely that glycolytic ATP simultaneously affects multiple effectors, we also simulated combinatorial effector mechanisms. To avoid bias, we varied, for each respective combination, the *k* values of each individual effector contributing to this combination by approximately a comparable extent (Supplementary Note). We identified *k* values for every effector combination that matched the experimental 1:1 and 9:1 PFKFB3^KD^:WT competition data (Fig. 2d,e; Supplementary Note Table 1).

These results show that the MSM-ATP is suited to simulate the PFKFB3^KD^ competition defect. Also, these simulations predicted that all three ATP-dependent effectors could mediate the role of PFKFB3-driven glycolysis in tip cell competition. We next evaluated another key phenotype of PFKFB3^KD^ ECs (impaired filopodia formation) to distinguish between the effector mechanisms (Supplementary Fig. 1).

### Regulation of filopodia formation by MSM-ATP effectors

**Figure 3:**
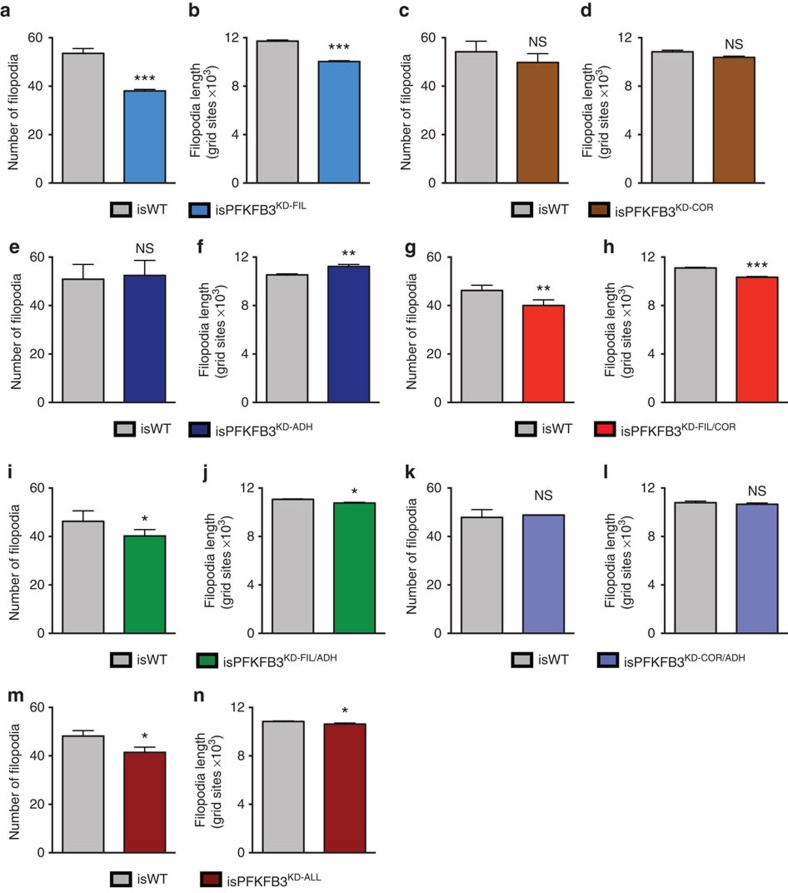
E^FIL^-containing isPFKFB3^KD^ mechanisms have reduced filopodia. (**a-n**) Number and length of filopodia for *in silico* WT (isWT) and *in silico* PFKFB3^KD^ (isPFKFB3^KD^) cells, modelled by modifying the single mechanisms E^FIL^ (**a**,**b**), E^COR^ (**c**,**d**) and E^ADH^ (**e**,**f**), and the combinatorial mechanisms E^FIL/COR^ (**g**,**h**), E^FIL/ADH^ (**i**,**j**), E^COR/ADH^ (**k**,**l**) and E^ALL^ (**m**,**n**). Data are mean±s.e.m.; *n*=4–5; NS, not significant, *P<0.05, **P<0.01, ***P<0.001; Student's *t*-test.

Intuitively, the E^FIL^ mechanism was expected to match the PFKFB3^KD^ filopodia defect. However, E^ADH^ and E^COR^ might also indirectly affect filopodia formation, since they affect intercellular junctions, and junction size determines DLL4/Notch lateral inhibition, which restricts filopodia formation^24^. While isPFKFB3^KD-FIL^ cells indeed formed fewer and shorter filopodia, isPFKFB3^KD-COR^ or isPFKFB3^KD-ADH^ cells formed filopodia normally (Fig. 3a–f, Supplementary Movies 2, 3, 4). We therefore discarded the single E^COR^ and E^ADH^ mechanisms, as they failed to recapitulate this essential trait of PFKFB3^KD^ ECs (Supplementary Fig. 1).

Among the combinatorial effector mechanisms, only those in which E^FIL^ was affected, matched the PFKFB3^KD^ filopodia defect (Fig. 3g–n, Supplementary Movies 5, 6, 7, 8). We therefore discarded E^COR/ADH^ and retained E^FIL^, E^FIL/COR^, E^FIL/ADH^ or E^ALL^ as potential mechanisms (Supplementary Fig. 1). We then studied whether we could further refine the search for the most plausible mechanism by focusing on intercellular adhesion and cortical protrusion formation, other potentially ATP-requiring processes.

### PFKFB3^KD^ increases intercellular heterogeneity

We recently reported that heterogeneous EC behaviour, based on differential VE-cadherin-dependent intercellular adhesion and differential formation of cortical protrusions, drives EC rearrangement during vessel sprouting^9^. Since the VEGFR2/Notch signalling axis steers these processes, inhibition of Notch signalling by the γ-secretase inhibitor DAPT removes all intercellular heterogeneity in a sprout and causes all ECs to become active and weakly adhesive^9^. Notably, PFKFB3 blockade reduces DAPT-induced vascular hypersprouting to normal levels^6^. We thus simulated DAPT-treated 1:1 isPFKFB3^KD^:isWT sprouts (modelled by removing Notch1-signalling between all cells) and studied whether the retained effector mechanisms promoted intercellular heterogeneity in the sprout.

**Figure 4:**
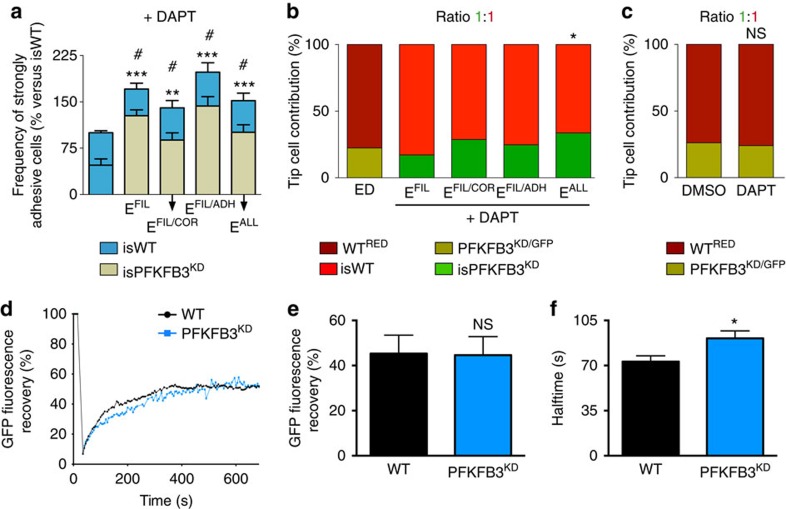
PFKFB3^KD^ increases intercellular heterogeneity. (**a**) Analysis of EC adhesive strength of isWT cells in isWT:isWT sprouts (first bar) and isPFKFB3^KD-FIL^, isPFKFB3^KD-FIL/COR^, isPFKFB3^KD-FIL/ADH^ and isPFKFB3^KD-ALL^ cells in a 1:1 isPFKFB3^KD^:isWT mosaic sprout treated with DAPT. Modifying E^FIL^, E^FIL/COR^, E^FIL/ADH^ and E^ALL^ increased the fraction of isPFKFB3^KD^ cells that were classified as strongly adhesive, less motile cells (resulting in more heterogeneity in adhesion between sprout cells). Data are mean±s.e.m.; *n*=10; **P<0.01 and ***P<0.001 versus isWT for total number of strongly adhesive cells; #*P*<0.001: frequency of strongly adhesive isPFKFB3^KD^ mutants versus the expected frequency (50%); Student's *t*-test. (**b**) E^FIL^, E^FIL/COR^, E^FIL/ADH^, and E^ALL^ simulations of tip cell contribution in a 1:1 isPFKFB3^KD^:isWT mosaic sprout treated with DAPT. The first bar shows the *in vitro* experimental data (ED, *n*=30 spheroids from 3 donors) obtained when using PFKFB3^KD^:WT sprouts. Only isPFKFB3^KD-ALL^ cells showed different tip cell behaviour upon DAPT treatment. *n*=150; *P<0.05 versus ED; Fisher's exact test. (**c**) *In vitro* EC spheroid assay using 1:1 mosaic PFKFB3^KD/GFP^:WT^RED^ sprouts treated with DAPT or control vehicle (DMSO), showing that DAPT treatment did not change the impaired tip cell contribution of PFKFB3^KD/GFP^ cells. *n*=70 spheroids from 3 donors; NS: not significant; Fisher's exact test. (**d-f**) FRAP experiment using confluent cultures of WT and PFKFB3^KD^ ECs expressing a VE-cadherin-GFP fusion protein. Representative fluorescence recovery curves after photobleaching (**d**). Quantification of VE-cadherin mobility (**e**) and turnover (**f**) for control and PFKFB3^KD^ ECs (*n*=27 (WT) and *n*=28 (PFKFB3^KD^) from 3 donors). Data are mean±s.e.m.; NS, not significant, *P<0.05; Student's *t*-test.

First, we evaluated whether differential VE-cadherin-dependent adhesion was increased in DAPT-treated isPFKFB3^KD^ cells. As VEGFR2 signalling determines VE-cadherin endocytosis^10^, we assessed VE-cadherin-dependent intercellular adhesion by determining the fraction of cells with low VEGFR2 activity (strongly adhesive cells). These simulations revealed that E^FIL^ and E^ADH^ increased adhesion, but E^COR^ did not (Fig. 4a; Supplementary Fig. 2a). Indeed, since lowering E^FIL^ reduced the number and length of filopodia (Fig. 3a,b), and filopodia amplify VEGF signalling by expressing VEGFR2 (ref. 22), it decreased VEGFR2 signalling in isPFKFB3^KD^ cells and thus increased adhesion. Moreover, increasing E^ADH^ reduced VE-cadherin endocytosis and therefore increased adhesion.

The number of strongly adhesive isPFKFB3^KD^ cells was consequently increased for each remaining mechanism (Fig. 4a). Thus, these cells had a disadvantage to reach the sprout's front as compared with the less-adhesive isWT cells, and therefore contributed less to the tip upon DAPT treatment, similar to the PFKFB3^KD^ competition defect in control conditions (Fig. 4b). However, isPFKFB3^KD-ALL^ cells contributed more to the tip than the cells of the other isPFKFB3^KD^ mechanisms (Fig. 4b), because one of their contributing effectors (E^COR^) did not increase adhesion and because the *k*_*FIL*_, *k*_*COR*_ and *k*_*ADH*_ values, obtained for isPFKFB3^KD-ALL^ cells to match the experimental data (Fig. 2d,e), were relatively low (Supplementary Note). Thus, filopodia formation and intercellular adhesion mediated the effects of PFKFB3-driven glycolysis on differential adhesion in the sprout, required for EC rearrangement.

Next, we focused on cortical protrusions, which are formed excessively when Notch signalling is inhibited by DAPT^9^. Lowering E^COR^ in isPFKFB3^KD-FIL/COR^ and isPFKFB3^KD-ALL^ cells reduced cortical protrusion formation in DAPT-treated 1:1 isPFKFB3^KD^:isWT sprouts (Supplementary Fig. 2b). Hence, modifying E^FIL/COR^ and E^ALL^ modestly increased the differential formation of cortical protrusions. In contrast, modifying E^FIL^ or E^FIL/ADH^ did not reduce cortical protrusion formation in DAPT conditions (Supplementary Fig. 2b). Since the reduction in cortical protrusion formation was similar in isPFKFB3^KD-FIL/COR^ and isPFKFB3^KD-ALL^ cells (Supplementary Fig. 2b) and as the competitiveness of isPFKFB3^KD-ALL^ cells was higher (Fig. 4b), cortical protrusion formation was not expected to contribute to the PFKFB3-driven effect on cellular heterogeneity in the DAPT-treated sprout.

To validate *in vitro* these computational predictions, we treated 1:1 PFKFB3^KD^:WT EC spheroids with DAPT. We observed that PFKFB3^KD^ ECs still contributed less to the tip position (Fig. 4c). Hence, only the isPFKFB3^KD-FIL^, isPFKFB3^KD-FIL/COR^ and isPFKFB3^KD-FIL/ADH^ mechanisms correctly predicted the experimental outcome. We thus discarded the isPFKFB3^KD-ALL^ mechanism and only retained isPFKFB3^KD-FIL^, isPFKFB3^KD-FIL/COR^ and isPFKFB3^KD-FIL/ADH^ as mechanisms that potentially explained the role of glycolytic ATP in EC rearrangement (Supplementary Fig. 1). Overall, the MSM-ATP predicted that PFKFB3-driven glycolysis affects vessel sprouting through effects on filopodia formation, alone or together with cortical protrusion formation or adhesion.

### PFKFB3^KD^ reduces VE-cadherin turnover

To validate the regulation of intercellular VE-cadherin-dependent adhesion by glycolytic ATP, we measured VE-cadherin expression and dynamics in cultured PFKFB3^KD^ ECs. PFKFB3^KD^ did not change VE-cadherin expression levels (Supplementary Fig. 2c). We thus quantified VE-cadherin mobility and turnover (that is, reappearance of VE-cadherin at the cell surface after prior endocytosis) at junctions by fluorescence recovery after photobleaching (FRAP). As the magnitude of fluorescence recovery was similar in PFKFB3^KD^ and WT ECs, PFKFB3^KD^ did not change the mobile VE-cadherin fraction in the plasma membrane (Fig. 4d,e). However, the fluorescence recovery rate was lower in PFKFB3^KD^ ECs, as revealed by the longer halftime, indicating that VE-cadherin turnover was reduced by PFKFB3^KD^ (Fig. 4d,f). These findings confirmed the *in silico* predicted role of VE-cadherin-dependent intercellular adhesion in the PFKFB3^KD^ phenotype.

### High VEGF levels perturb EC rearrangement

We then simulated pathological angiogenesis to explore via which effector mechanisms PFKFB3 affected vessel sprouting in non-physiological conditions, given that PFKFB3 blockade inhibits ocular and inflammation-induced neovascularization^6^,^7^. Dysfunctional EC rearrangement is a feature of pathological angiogenesis, as demonstrated in mouse models characterized by high VEGF levels such as oxygen induced retinopathy and cancer^9^,^28^. Before exploring whether PFKFB3 blockade normalized EC rearrangements in pathological conditions, we simulated a 2-fold (‘2x VEGF') and 10-fold (‘10x VEGF') increase in VEGF concentrations in the MSM-ATP. We selected these conditions since VEGF levels in tumours generally increase by 2- to 10-fold in early and late stages of tumour progression^29^,^30^,^31^,^32^, and analysed cell shuffling and the S&P pattern formation of isWT sprouts during 24 h of sprouting.

We assessed cell shuffling by tracking the number of cell–cell overtakes and the duration that individual cells spend at the sprout's tip (with respect to their initial position). The sprout's ability to acquire and maintain a S&P pattern of activated and inhibited cells reflects DLL4/Notch-mediated signalling (Supplementary Fig. 3a, Supplementary Movie 9). We therefore developed a new method of scoring S&P patterns that accounted for their dynamic nature, since cells in the sprout shuffle and change neighbours, continuously disrupting and re-establishing S&P patterns. We assessed the average and maximal time that a S&P pattern was maintained, as well as the intermittent time between subsequent S&P patterns, called the stabilizing time.

Similar to previous observations with the MSM^22^,^24^, we found using the MSM-ATP that the higher the VEGF levels were, the more synchronized and oscillatory the cells of an isWT sprout became. This implies that the cells were all active or inhibited, rather than organized in an alternating S&P pattern (Supplementary Fig. 3b,c; Supplementary Movie 10). This abnormal behaviour was reflected by: (i) a lower number of cell–cell overtakes (Supplementary Fig. 3d, Supplementary Movies 11); (ii) a decreased ability to form and maintain a S&P pattern (Supplementary Fig. 3e–g); (iii) reduced cell rearrangement in the sprout (Supplementary Fig. 3h–j); and consistent herewith (iv) a reduced chance for rear cells to reach the tip position (Supplementary Fig. 3k). We used these results in high VEGF conditions to assess the effects of *in silico* pharmacological PFKFB3 blockade on this dysfunctional EC rearrangement, and to evaluate whether normalization of EC rearrangement might underlie the beneficial effects observed upon *in vivo* pharmacological PFKFB3 blockade^6^. We therefore explored if any of the three residual effector mechanisms could normalize the perturbed EC rearrangement in pathological conditions.

### PFKFB3 blockade is predicted to normalize EC rearrangement

We first re-calibrated the MSM-ATP to ensure that it matched the impaired *in vitro* EC migration and sprouting upon PFKFB3 blockade by 3-(3-pyridinyl)-1-(4-pyridinyl)-2-propen-1-one (3PO) or 7,8-dihydroxy-3-(4-hydroxyphenyl)-chromen-4-one^6^ (YN1; Supplementary Note Table 2). Since pharmacological drugs indiscriminately target all cells of an angiogenic sprout, we modelled *in silico* pharmacological PFKFB3 inhibition (isPFKFB3^PI^) by using non-mosaic sprouts in which all cells experience PFKFB3 inhibition.

We then assessed whether any of the remaining effector mechanisms (isPFKFB3^PI-FIL^, isPFKFB3^PI-FIL/COR^, isPFKFB3^PI-FIL/ADH^) could reverse the abnormal EC behaviour in high VEGF conditions. Before simulating the (very) high VEGF conditions (as observed in tumours), we confirmed the accuracy of our modelling approach by simulating a 1.44-fold increase in VEGF levels, reflecting the increase in VEGF in choroid neovascularization (CNV)^33^. All aforementioned EC perturbations were completely normalized, except for the positional interchanges in isPFKFB3^PI-FIL/COR^ sprouts (Supplementary Fig. 4a–e). Hence, these results suggest that a normalized EC rearrangement seemingly underlies the reduced pathological angiogenesis upon PFKFB3 blockade in the CNV model^6^.

**Figure 5:**
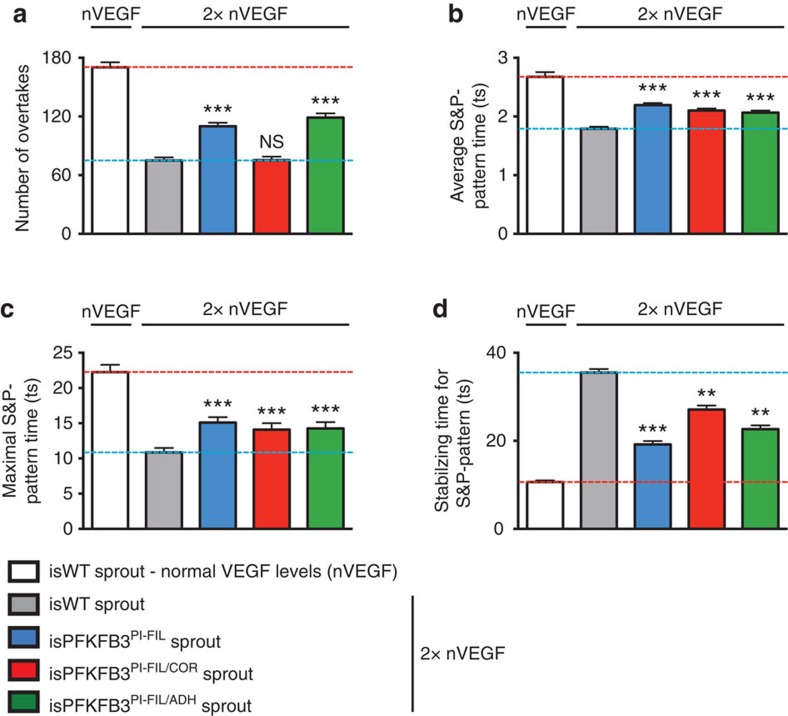
Modifying E^FIL^ and E^FIL/ADH^ partially normalizes EC dynamics. (**a–d**) Number of overtakes (**a**), the average (**b**) and maximal time (**c**) during which a salt and pepper (S&P) pattern is maintained, and the time required to acquire a stable S&P pattern (**d**) for *in silico* WT (isWT) ECs in non-mosaic sprouts, in which PFKFB3 was pharmacologically blocked (isPFKFB3^PI^) simulated by modifying E^FIL^ (blue), E^FIL/COR^ (red) and E^FIL/ADH^ (green) in VEGF levels 2-fold higher than normal (2 × nVEGF). The white and grey bars represent a simulated isWT sprout in normal VEGF (nVEGF) and 2 × nVEGF levels, respectively. The horizontal red and blue dotted lines show the particular values of an isWT sprout in normal and 2 × VEGF levels, respectively. ts: timestep. Data are mean±s.e.m.; *n*=50; NS, not significant, **P<0.01, ***P<0.001 versus isWT in 2 × nVEGF; Student's *t*-test.

When studying 2 × VEGF levels, we observed that the abnormal EC features were partially normalized in an isPFKFB3^PI-FIL^ sprout. Indeed, isPFKFB3^PI-FIL^ cells, positioned initially at the rear of the sprout, had a higher chance of reaching the front (Supplementary Fig. 5a) and more frequently overtook neighbouring cells (Fig. 5a). In addition, by reducing filopodia formation, lowering E^FIL^ impaired the responsiveness to VEGF and thereby increased the Notch-driven heterogeneity between isPFKFB3^PI-FIL^ cells (Fig. 5b–d). This counteracted the perturbed S&P patterning, explaining why isPFKFB3^PI-FIL^ cells overall had an increased ability to reach and maintain stable S&P patterns.

Similar findings were obtained for isPFKFB3^PI-FIL/ADH^ cells (Fig. 5a–d; Supplementary Fig. 5a). However, the stabilizing time for these cells was reduced less than for isPFKFB3^PI-FIL^ cells (Fig. 5d). This was likely due to the lower *k*_*FIL*_ value used to match the PFKFB3^KD^:WT EC competition data (Fig. 2d,e). Qualitatively, similar findings regarding the ability to form and maintain S&P patterns were obtained for isPFKFB3^PI-FIL/COR^ cells (Fig. 5b-d), though the stabilizing time for these cells was reduced the least (Fig. 5d). However, reducing E^COR^ in addition to E^FIL^ counteracted the normalization effect of reducing E^FIL^ on the overtaking and cell shuffling behaviour (Fig. 5a; Supplementary Fig. 5a) by impairing EC migration (Supplementary Fig. 5b). Thus, the E^FIL^ and E^FIL/ADH^ mechanisms normalized the abnormal EC rearrangement the best. In contrast, the E^FIL^, E^FIL/COR^ and E^FIL/ADH^ mechanisms failed to normalize the abnormal EC features in 10 × VEGF levels (Supplementary Fig. 6a–e). In fact, isPFKFB3^PI-FIL/COR^ sprouts showed even an increased, albeit not significantly, stabilizing time and a decreased number of overtakes, thus further disorganizing rather than normalizing EC behaviour.

Overall, the prediction that EC disorganization was normalized only minimally for isPFKFB3^PI-FIL/COR^ sprouts excluded E^FIL/COR^ as a plausible mechanism to explain the PFKFB3^KD^ phenotype, since PFKFB3 blockade reduces pathological angiogenesis^6^. Hence, taken the results of all *in silico* and *in vitro* experiments together, the mechanisms that best explain the PFKFB3^KD^ phenotype across all conditions are E^FIL^ and E^FIL/ADH^. However, since the VE-cadherin turnover was reduced upon PFKFB3^KD^ in the FRAP experiment (Fig. 4d-f), E^FIL/ADH^ is the most likely mechanism to reproduce the complete PFKFB3^KD^ phenotype. While previous studies documented a role for filopodia in tip cell selection^22^ and for PFKFB3-driven glycolysis in filopodia formation by tip cells^5^, this study identified a previously unknown role for PFKFB3-driven glycolysis in filopodia formation and intercellular adhesion underlying EC rearrangement during vessel sprouting.

### Combined PFKFB3/VEGF blockade normalizes vessel sprouting

**Figure 6:**
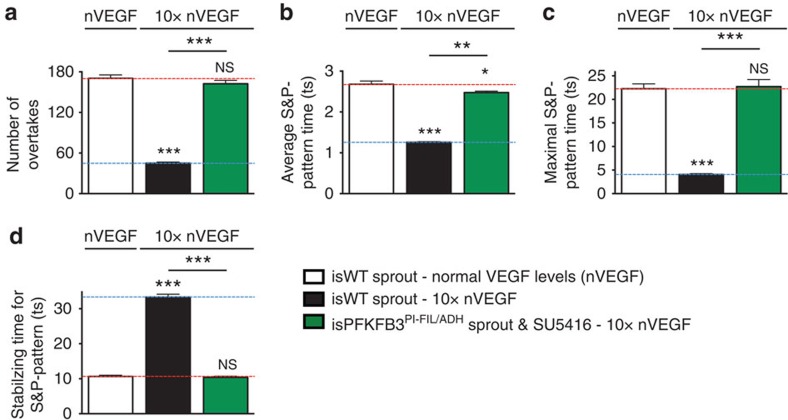
Anti-VEGF is predicted to synergize with anti-metabolic therapy. (**a–d**) Number of overtakes (**a**), the average (**b**) and maximal time (**c**) during which a salt and pepper (S&P) pattern is maintained, and the time required to acquire a stable S&P pattern (**d**) for *in silico* WT (isWT) ECs in non-mosaic sprouts, in which PFKFB3 was pharmacologically blocked (isPFKFB3^PI^) simulated by modifying E^FIL/ADH^ and blocking VEGF signalling (green; ‘isPFKFB3^PI-FIL/ADH^ sprout and SU5416') in VEGF levels 10-fold higher than normal (10 × nVEGF). The white and black bars represent a simulated isWT sprout in normal VEGF (nVEGF) and 10 × nVEGF levels, respectively. The horizontal red and blue dotted lines show the particular values of an isWT sprout in normal and 10x VEGF levels, respectively. ts, timestep. Data are mean±s.e.m.; *n*=50; NS, not significant, ***P<0.001 versus isWT in nVEGF; the statistical significance between the black and green bars is also indicated (**P<0.01, ***P<0.001); Student's *t*-test.

Since VEGF signalling regulates E^FIL^ and E^ADH 9^, and 3PO enhances the anti-angiogenic effect of a VEGF receptor kinase inhibitor (SU5416) in physiological conditions^6^, we assessed *in silico* whether blockade of both PFKFB3 and VEGF signalling might be more efficient in normalizing the deregulated EC rearrangement in pathological conditions (10 × VEGF). Since SU5416 inhibits VEGFR2 phosphorylation^34^, we modelled the anti-VEGF therapy by reducing VEGFR2 signalling activity (Supplementary Note). This simulation predicted that an anti-VEGF and anti-glycolytic treatment together were most effective in normalizing EC behaviour (Fig. 6a–d; Supplementary Fig. 7a, Supplementary Movies 12, 13). A full rescue with the combined treatment was observed when the VEGFR2 signalling activity was decreased sixfold. Applying anti-VEGF therapy alone in 10x VEGF levels was predicted to only partially normalize the disorganized EC dynamics (Supplementary Fig. 7b-e). Hence, the simulations predicted that combinatorial anti-glycolytic and anti-VEGF treatment would normalize EC rearrangement in disease.

**Figure 7:**
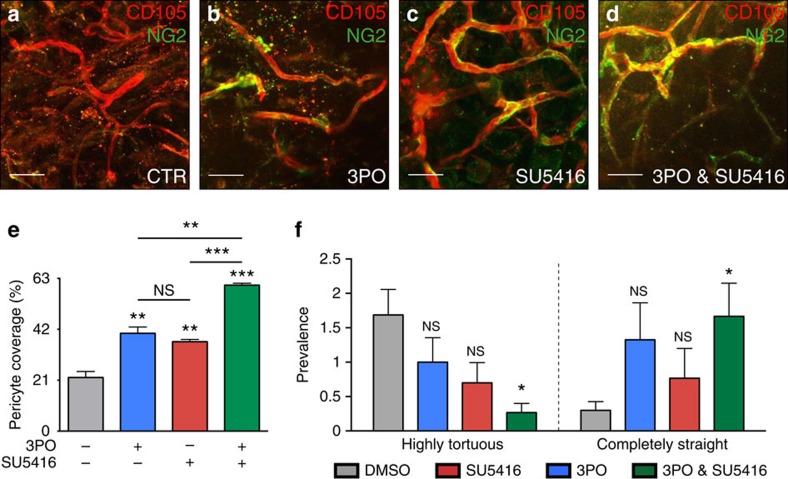
3PO plus SU5416 combo-therapy normalizes pathological vessels. (**a–d**) Representative images of VEGF-containing Matrigel plugs treated with control vehicle (**a**), the PFKFB3 blocker 3PO (**b**), the VEGFR2 inhibitor SU5416 (**c**), and a combination treatment of 3PO plus SU5416 (**d**), and immunostained for the EC marker CD105 (red) and the pericyte marker NG2 (green). Scale bar, 40 μm. (**e**) Quantification of the NG2-coverage of vessels in the Matrigel model, treated with control vehicle (DMSO, grey), 3PO (blue), SU5416 (red), or 3PO plus SU5416 (green). Data are mean±s.e.m.; *n*=3–5 Matrigel plugs per condition; NS: not significant, **P<0.01, ***P<0.001; Student's *t*-test. (**f**) Blood vessel tortuosity in the Matrigel model, treated with control vehicle (DMSO, grey), 3PO (blue), SU5416 (red), or 3PO plus SU5416 (green), was analysed semi-quantitatively by an observer blinded for the conditions. Data are mean±s.e.m.; *n*=3–5 Matrigel plugs per condition; NS, not significant; *P<0.05; Two-tailed Wilcoxon rank-sum test for equal medians.

To validate this prediction experimentally, we tested whether PFKFB3 blockade, as monotherapy and as combination therapy with VEGF blockade, could normalize pathological vessels in two established models of vessel disorganization, characterized by structurally abnormal vessels with deregulated EC rearrangements: (i) Matrigel plugs containing high amounts of VEGF^35^, and (ii) retinas of postnatal day 5 (P5) mouse pups upon intraocular injection of high VEGF amounts^9^,^28^. In the Matrigel model, high VEGF levels induce the formation of structurally abnormal and disorganized vessels, characterized by tortuosity and poor pericyte coverage^35^. Monotherapy with 3PO or SU5416 improved the association between pericytes and ECs (vessel maturation, a sign of vessel normalization^36^) (Fig. 7a-c,e), and tended to reduce vessel tortuosity (Fig. 7f). However, these vessel normalization effects were most prominent and statistically robust upon combined treatment with 3PO plus SU5416 (Fig. 7d-f).

**Figure 8:**
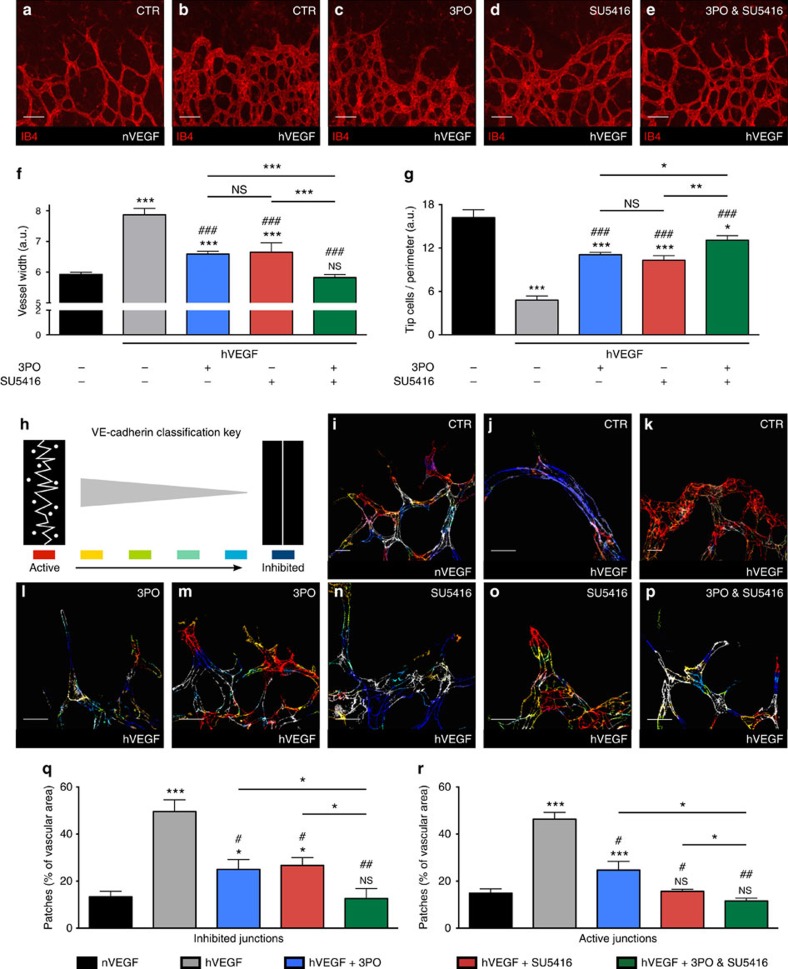
3PO / SU5416 combo-therapy normalizes pathological angiogenesis. (**a–e**) Representative images of retinas (× 20 magnification) stained for isolectinB4 (IB4) in normal conditions (nVEGF, a) and after VEGF injection (hVEGF, b-e). Pups were treated with DMSO (CTR, **a**,**b**), 3PO (**c**), SU5416 (**d**) or 3PO plus SU5416 (**e**). Note the widened vessel lumen and fewer tip cells at the vascular forefront upon VEGF injection (**b**), and the partial (**c,d**) and complete (**e**) normalization by single or combined 3PO and SU5416 treatment. Scale bar, 50 μm. (**f,g**) Quantification of vessel width (f) and tip cell number at the retinal front (g). Data are mean±s.e.m.; *n*=7 per condition; NS: not significant, *P<0.05, ***P<0.001 versus DMSO-treated vessels in normal conditions. ###, *P*<0.001 versus DMSO-treated vessels in hVEGF. The statistical significance between the single or combined 3PO/SU5416 treatments is also indicated (NS: not significant, *P<0.05, **P<0.01, ***P<0.001); Student's *t*-test. a.u., arbitrary units. (**h**) VE-cadherin junction classification from ‘active' (red; irregular/serrated morphology with vesicular/diffuse regions) to ‘inhibited' (dark blue; straighter morphology and less vesicular staining). (**i–p**) VE-cadherin morphology of retinal vessels was hand-classified according to the key in h, yielding VE-cadherin heat maps in normal conditions (nVEGF, **i**) and upon VEGF injection (hVEGF, **j**–**p**) of pups, treated with DMSO (**j,k**), 3PO (**l,m**), SU5416 (**n,o**), or 3PO plus SU5416 (**p**). The colour(s) provide insight into the extent of junctional heterogeneity of the vessels. Compared to the heterogeneous VE-cadherin pattern in nVEGF retinas (**i**), hVEGF induces clusters of inhibited (**j**) or activated (**k**) ECs. These clusters are smaller upon 3PO (inhibited: **l**; active: **m**) or SU5416 monotherapy (inhibited: **n**; active: **o**) and completely normalized to heterogeneous patterns upon combined 3PO plus SU5416 treatment (**p**). Scale bar, 20 μm. (**q,r**) Prevalence of strongly inhibited (**q**) or activated (**r**) VE-cadherin junctions in retinal vessels. Data are mean±s.e.m.; *n*=5–9 per condition; NS, not significant, *P<0.05, ***P<0.001 versus nVEGF (black); #*P*<0.05, ##*P*<0.01 versus hVEGF (grey). The statistical significance between the single or combined 3PO/SU5416 treatments is also indicated (*P<0.05); One-tailed Wilcoxon rank-sum test for equal medians.

In the retina model, intraocular VEGF injection evokes marked structural vessel abnormalities and deregulated EC rearrangements, and has therefore become an increasingly used model to study these processes^9^,^23^,^28^,^37^. Indeed, intraocular VEGF injection evokes vessel expansion^28^, reduces the number of tip cells at the vascular front (a consequence of disrupted DLL4/Notch signalling)^28^, and changes the differential VE-cadherin pattern at EC junctions so that clustered regions are formed, within which ECs are either active or inhibited^9^, all signs of EC rearrangement defects^9^,^26^. VEGF indeed enlarged vessel size and decreased the number of tip cells (Fig. 8a,b,f,g). Monotherapy with 3PO or SU5416 partially restored the vessel width and the number of tip cells (Fig. 8c,d,f,g), but the largest normalization effect was obtained with the combination of 3PO plus SU5416 (Fig. 8e-g). We then quantified the VE-cadherin pattern at individual EC junctions using established image analysis software^9^, classifying junctions in a graded scale from ‘active' (irregular, serrated junctions with vesicular, diffuse appearance) to ‘inhibited' (with a straighter and less vesicular morphology) (Fig. 8h). Injection of VEGF reduced the VE-cadherin junctional heterogeneity, inducing the formation of clusters containing either active or inhibited ECs (Fig. 8i-k,q,r). Monotherapy with 3PO or SU5416 partially normalized the VE-cadherin junctional pattern (becoming more heterogeneous again, but still containing small clustered regions of active or inhibited junctions) (Fig. 8l-o,q,r), while the combination treatment with 3PO plus SU5416 completely normalized the VE-cadherin junctional heterogeneity (Fig. 8p-r). Taken together, these *in vivo* results validated the *in silico* predictions.

## Discussion

Through an integrated combination of computational and experimental approaches, we identified the combinatorial E^FIL/ADH^ mechanism as the only mechanism that matches the endothelial PFKFB3^KD^ phenotype across all conditions. PFKFB3^KD^ thus reduces EC rearrangement and tip cell competition by increasing adhesion and reducing filopodia formation. This previously unknown regulation of intercellular adhesion by PFKFB3 was validated experimentally. Hence, through modelling, we obtained a mechanistic explanation for the reduced tip cell competitiveness of PFKFB3^KD^ ECs. In addition to cytoskeletal remodelling leading to filopodia formation, glycolysis also regulates adhesion in sprouting ECs. Uncovering such regulation of closely linked individual mechanisms by PFKFB3 is difficult to achieve experimentally, conditions in which computational modelling can greatly assist experimental research^38^. Moreover, the model predicts that treatment with a PFKFB3 blocker normalizes perturbed EC rearrangements in pathological conditions. We verified this prediction in two established models of vessel disorganization, and observed that PFKFB3 blockade indeed evoked normalization of EC rearrangements during pathological angiogenesis *in vivo*.

We previously documented that PFKFB3^KD^ impairs filopodia formation of tip cells^5^. However, we did not characterize the mechanism via which this filopodia defect regulated vessel sprouting. Here we show that reduced formation of filopodia upon PFKFB3^KD^ has a substantial impact on EC rearrangement during vessel sprouting. Indeed, isPFKFB3^KD-FIL^ ECs extend fewer and shorter filopodia, thereby affecting VEGFR2 activation and shifting the cells to a more adhesive phenotype. Hence, isPFKFB3^KD-FIL^ ECs matched three key experimentally observed defects of PFKFB3^KD^ ECs, that is, impaired tip cell competition, reduced filopodia formation, and decreased pathological angiogenesis.

In addition, the simulations predicted unknown effects of reducing filopodia in high VEGF conditions, such as the (partial) normalization of the perturbed EC rearrangement and signalling dynamics. Indeed, reducing E^FIL^ increased the number of cell-cell overtakes and the likeliness of the rear cells to reach the tip position. Moreover, since filopodia act as signalling sensors that amplify lateral inhibition, reducing E^FIL^ in high VEGF conditions impaired the EC responsiveness to VEGF and thereby increased the Notch-driven intercellular heterogeneity. The latter mechanism counteracts the perturbed S&P patterning in high VEGF conditions, explaining why isPFKFB3^PI-FIL^ cells overall had an increased ability to reach and maintain stable S&P patterns.

Our simulation studies unveiled an unknown regulation by PFKFB3-driven glycolysis of intercellular adhesion, which is essential for EC rearrangement. In an isPFKFB3^KD-ADH^:isWT sprout, the weakly adhesive isWT cells had an advantage, while the strongly adhesive isPFKFB3^KD-ADH^ cells had a disadvantage to propel to the sprout's front. Hence, isPFKFB3^KD-ADH^ ECs matched a key phenotypic defect of PFKFB3^KD^ ECs, namely impaired tip cell competition. In contrast, filopodia formation was not affected. In high VEGF conditions, reducing E^ADH^ together with E^FIL^ also partially normalized the perturbed EC rearrangement. This unexpected glycolytic regulation of intercellular adhesion in a vessel sprout was experimentally validated by our findings that PFKFB3^KD^ lowered VE-cadherin turnover at the cell surface of cultured contacting ECs.

How PFKFB3-driven glycolysis regulates VE-cadherin adhesion remains to be determined, but we can speculate about possible mechanisms. First, PFKFB3-driven glycolysis generates ATP, which can be used to phosphorylate VE-cadherin before endocytosis^10^, thus reduced ATP availability would result in longer VE-cadherin exposure at the cell surface. Second, VE-cadherin and the actin cytoskeleton are physically connected via multiple adaptor proteins^39^,^40^. Since PFKFB3 controls actin cytoskeleton remodelling^5^ and endocytosis requires actin polymerization^41^, PFKFB3 might influence VE-cadherin dynamics via actin.

The simulations in pathological conditions predicted that blocking PFKFB3, via effects on filopodia formation and intercellular adhesion, was capable of completely or partially normalizing the perturbed EC rearrangement *in silico*, when VEGF levels were elevated 1.44- and 2-fold, respectively. This is in close agreement with findings that PFKFB3 blockade or loss in ECs can inhibit pathological angiogenesis in ocular, inflamed and malignant tissues^6^,^7^. Interestingly, the *in silico* CNV results suggest that normalized EC dynamics might contribute to the anti-angiogenic effect of PFKFB3 inhibition.

When vessels grow in pathological conditions, they are often structurally highly abnormal, a phenomenon that has been best studied in tumours, but is also relevant in ocular disease and atherosclerosis^36^,^42^. Disorganized vessels are dilated and tortuous, are covered by fewer pericytes, and are lined by an irregular endothelium, characterized by hypermotile ECs with excessive filopodia and deregulated EC rearrangements. Vessel normalization is therefore emerging as a therapeutic paradigm to restore tissue performance by improving vessel formation, function and interaction with the microenvironment^36^,^42^. We therefore simulated PFKFB3 blockade also in more extreme VEGF levels (10 × VEGF), corresponding with late-stage tumours. However, *in silico* PFKFB3 or VEGFR2 blockade as monotherapy was unable to completely normalize the perturbed EC rearrangements in these conditions. This required a combinatorial blockade of PFKFB3 plus VEGF signalling. Using established models of vessel disorganization, we experimentally validated these predictions *in vivo* by showing that a combination treatment with 3PO plus SU5416 reduced vessel dilatation and the numbers of tip cells at the vascular forefront, and restored differential junctional patterning in high VEGF-treated retinas. Moreover, we showed that this combined treatment normalized vessel tortuosity and pericyte coverage in VEGF-containing Matrigel plugs.

Our findings might be relevant in light of the emerging results that intrinsic refractoriness and acquired drug resistance limit the success of VEGF-targeted therapy in cancer and other diseases^42^. As tumour vessels have weak and disorganized VE-cadherin junctions^9^,^43^, strengthening these junctions by PFKFB3 plus VEGF blockade would normalize the endothelium and tighten the endothelial barrier, thereby constituting a stronger physical barrier for cancer cells to escape. In addition, our findings may also have implications for other disease conditions characterized by disorganized blood vessels in general, such as in ocular neovascularization, atherosclerosis and so on. Hence, our simulations were not only instrumental in identifying cellular mechanisms whereby PFKFB3-driven glycolysis regulates vessel sprouting, but also predicted vessel normalization by targeting EC metabolism (glycolysis).

## Methods

### Cell culture

Human umbilical venous endothelial cells (HUVECs, referred to as ‘ECs') were isolated from umbilical cords of different donors^44^. Umbilical cords were obtained from the Gynaecology Department of the University Hospital Leuven, with approval of the Medical Ethical Commission of KU Leuven/University Hospital Leuven, and informed consent was obtained from all subjects. The umbilical vein was rinsed with 20 ml DPBS (Gibco, Invitrogen, Life Technologies, Ghent, Belgium) and subsequently incubated with 20 ml warm collagenase type 1 (2 mg ml^−1^ in 0.9% NaCl supplemented with 2 mM CaCl_2_ and 1 × antibiotics/antimycotics; Gibco, Invitrogen, Life Technologies) for 15 min at 37 °C. The collagenase and ECs were collected and the vein was rinsed two times with EC culture medium. The collected fluid was filtered (40 μm nylon cell strainer, BD Pharmingen 555289, Erembodegem, Belgium), centrifuged at 800 r.p.m. for 5 min and resuspended in 15 ml culture medium. The isolated ECs were used between passage 1 and 5 and cultured in: (i) M199 medium (1 mg ml^−1^ D-glucose; Gibco, Invitrogen, Life Technologies) supplemented with 20% fetal bovine serum (Gibco, Invitrogen, Life Technologies), 2 mM L-glutamine, 30 μg l^−1^ endothelial cell growth factor supplements, 10 units ml^−1^ heparin (Sigma-Aldrich, Bornem, Belgium), 100 IU ml^−1^ penicillin and 100 μg ml^−1^ streptomycin, or (ii) endothelial cell growth medium 2 (ECGM-2; Promocell, Germany) supplemented with ECGM-2 SupplementMix C-39216 (Promocell, Germany). ECs were cultured at 37 °C and 5% CO_2_ and the growth medium was changed every 48 h.

### Lentiviral transductions

For all spheroid experiments, PFKFB3-targeting oligonucleotides (shPFKFB3) cloned into the pLVX-shRNA2 vector (No. PT4052-5; Clontech, Westburg BV, Leusden, The Netherlands) were used. A pRRL-mCherry Red vector, kindly provided by M. Mazzone, was used to fluorescently mark WT HUVECs. The full-length mouse VE-cadherin complementary DNA (cDNA) fused to the enhanced green fluorescent protein (GFP) cDNA was kindly provided by D. Vestweber (Max Planck Institute, Muenster, Germany) and was cloned into the pRRLsin.PPT.CMV.MCS MM.Wpre lentiviral vector (pLenti-MP2; Addgene). For PFKFB3 knockdown in the FRAP experiments, shPFKFB3 or scrambled control oligonucleotides cloned into the PLKO.1-vector (Sigma-Aldrich) were used. Lentiviruses were produced by transfection into 293T cells^45^. HUVECs were co-transduced with VE-cadherin-GFP virus and shPFKFB3- or scrambled short hairpin RNA (shRNA)-encoding PLKO virus. All lentiviruses were used at a multiplicity of infection of 20. HUVECs were transduced overnight in the presence of 0.5 μg ml^−1^ polybrene (Sigma-Aldrich), washed three times and replenished with fresh medium the next day. Transduced HUVECs were used in functional assays at least 3–4 days post transduction.

### EC spheroid assay

1,000 transduced and/or control ECs were cultured overnight as hanging drops in a 1:1 ratio in ECGM2 medium supplemented with 20% methyl cellulose (Sigma-Aldrich) to form spheroids. The resulting spheroids were embedded in a collagen matrix by overlaying them with 1 vol methyl cellulose containing 40% fetal bovine serum and subsequent addition of 1/3 vol NaHCO_3_ (15.6 mg ml^−1^), 4/5 vol collagen (collagen type 1, rat tail extract, Merck Millipore, Overijse, Belgium) and 2% vol NaOH (1M)^46^. The collagen solution containing spheroids was aliquoted in 24-well plates and allowed to polymerize for 30 min at 37 °C. Next, ECGM2 medium containing the γ-secretase inhibitor N-[N-(3,5-Difluorophenacetyl)-L-alanyl]-S-phenylglycine *t*-butyl ester (DAPT, 10 μM; Merck Millipore, Overijse, Belgium) or the corresponding control vehicle (dimethyl sulfoxide (DMSO), Sigma-Aldrich) was added. Finally, 24 h after the addition of the culture medium, the EC spheroids were fixed by adding an equal volume of 4% paraformaldehyde at room temperature for 30 min. Tip cell contribution of WT (red) or shRNA-transduced ECs (green) was manually analysed under a Zeiss Axiovert 40 CFL fluorescence microscope (× 40 objective) (Carl Zeiss, Munich, Germany) (see also below).

### FRAP analysis of HUVECs

ECs, co-transduced with lentiviral vectors expressing VE-cadherin-GFP and shPFKFB3/scrambled shRNA, were seeded at a density of 100,000 cells per well into a eight-well μ-slide (Ibidi 80826, Ibidi, Beloeil, Belgium), coated with 0.1% gelatin (Sigma-Aldrich), for imaging on the next day. Live imaging and photobleaching of VE-cadherin-GFP-positive ECs was performed using a Zeiss LSM 780 (Axio Observer.Z1) confocal microscope (Carl Zeiss, Munich, Germany) at × 100 magnification while using an incubation chamber set at 37 °C and 5% CO_2_. Before photobleaching, four Z-stack images were acquired. Then the GFP-fluorophore was bleached for 150 iterations using a 488-nm argon laser at 100% laser power. Images were acquired at 135 acquisitions every 5 s for a period of 10–13 min using the Definite Focus System. Fluorescence intensities were analysed using the ZEN microscope software version 2012 (Carl Zeiss). The values of fluorescence intensities after photobleaching were normalized with the fluorescence intensity before photobleaching and subsequently analysed in Graphpad Prism v6.0f by performing a non-linear regression with one-phase association. Only recovery curves with a *R*^2^ value >0.95 were taken into consideration.

### RNA analysis

RNA expression analysis was performed by Taqman quantitative reverse transcriptase–PCR, which was read in MicroAmp Fast Optical 96-well Reaction Plates (Applied Biosystems, Carlsbad, CA). The cDNA (2.5 ng μl^−1^), the Taqman Fast Universal PCR Master Mix (Applied Biosystems) and the premade primer mix with probe (final concentration of primers and probe were 900 and 250 nM, respectively; Applied Biosystems and IDT, Leuven, Belgium) were mixed on ice. Primer ID numbers were Hs.PT.56a.4732035 (VE-cadherin) and Hs.PT.58.2145446 (HPRT). The samples were measured with the 7500 Fast Real-Time PCR system (Applied Biosystems), using the following cycling conditions: 20 s 95 °C, 40 cycles of 95 °C for 3 s and 30 s 60 °C.

### Animals

C57BL/6J or C57BL/6N mice were obtained from the KU Leuven Animal Facility or were purchased from Charles River (Wilmington, MA, USA). Animal procedures were approved by the Institutional Animal Care and Research Advisory Committee (KU Leuven) and were performed in accordance with the institutional and national guidelines and regulations.

### Matrigel model of *in vivo* angiogenesis

Growth factor-reduced Matrigel (Corning, New York, USA; 500 μl) supplemented with 200 ng ml^−1^ VEGF-A 165 (VIB Protein Service Facility), 500 ng ml^−1^ bFGF (Peprotech, London, UK) and 60 U ml^−1^ heparin (Leo Pharma, Wilrijk, Belgium) was subcutaneously injected on the back of C57BL/6J mice (protocol adapted from ref. 35). Pathological vasculature was allowed to develop during 6 days, followed by 5 consecutive days of treatment by intraperitoneal injection of control vehicle (DMSO, Merck Millipore, Overijse, Belgium), 3PO (ChemBridge Corporation, San Diego, USA; 25 mg kg^−1^ per day) and/or the VEGFR2 tyrosine kinase inhibitor SU5416 (Sigma-Aldrich; 10 mg kg^−1^ per day). These doses of 3PO and SU5416 were determined in pilot dose-response experiments. Three to five mice were used per group. Matrigel plugs were collected and stained for CD105 and NG2 to analyse vessel tortuosity and pericyte coverage.

### Postnatal retinal angiogenesis model

300 ng VEGF-A 165 (0.5 μl; VIB Protein Service Facility) was injected into the eyes of P5 C57BL/6N pups using a Hamilton micro-syringe^9^,^23^. Subsequently, the pups were treated by intraperitoneal injection of control vehicle (DMSO, Merck Millipore, Overijse, Belgium), 3PO (ChemBridge Corporation, San Diego, USA; 25 mg kg^−1^) and/or the tyrosine kinase inhibitor SU5416 (Sigma-Aldrich; 12 mg kg^−1^). Seven pups were used per group. These doses of 3PO and SU5416 were determined in pilot dose-response experiments. Twenty hours after VEGF injection, pups were euthanized, eyes were enucleated and processed for isolectinB4 and VE-cadherin staining.

### Immunohistochemistry

Matrigel plugs were stained using the primary antibodies anti-CD105/endoglin (1:50, R&D Systems, Minneapolis, MN, USA) and anti-neural/glial antigen 2 (NG2, 1:200; Chemicon-Millipore, Merck, Germany) to characterize ‘tumour' vessels. Matrigel plugs were then incubated with the appropriate fluorescently conjugated secondary antibodies (Alexa 568 or 647, 1:200, Molecular Probes, Invitrogen, Life Technologies). The Matrigel plugs were imaged at a magnification of × 20 using a Zeiss LSM 780 (Axio Observer.Z1) confocal microscope (Carl Zeiss). Retinas were collected on P6 and fixed with 4% paraformaldehyde for 1 h at room temperature. They were subsequently stained using the anti-VE-cadherin antibody^9^ (1:25, BD Pharmingen 555289, Erembodegem, Belgium) and isolectinB4-Alexa antibody (1:100, Molecular Probes, Invitrogen, Life Technologies)^5^. The secondary antibody targeting the anti-VE-cadherin antibody was the appropriate fluorescently conjugated Alexa 488 antibody (1:500, Molecular Probes, Invitrogen, Life Technologies). Retinas were imaged at magnification × 20 and × 63 using a Zeiss LSM 780 (Axio Observer.Z1) confocal microscope (Carl Zeiss).

### VE-cadherin junctional activity

VE-cadherin junctional activity of retinas was analysed by hand using the patching algorithm^9^. The algorithm processed each optical plane of the z-stack of the confocal retinal images (× 63 magnification) into random patches of 100 × 100 pixels based on a user-defined threshold for the anti-VE-cadherin signal. The resulting patches were subsequently blindly presented to the user who hand-classified the VE-cadherin morphology in each patch according to the key shown in Fig. 8h. Next, the classified patches were used to reconstruct images as three-dimensional-summed z-projections of the patch class colour overlaid on the VE-cadherin staining. The resulting colour(s) give insight into the extent of junctional heterogeneity of the vessels.

### Vessel width of retinal blood vessels

To analyse the vessel width of the retinal front, we imaged isolectinB4-stained retinas at a magnification of × 5 using a Leica DMI6000B inverted microscope (Leica Microsystems, Mannheim, Germany) and measured the distance from the centre of the dissected retina to the retinal front. The upper third of this distance of the retina lobes was subsequently used for analysis. Vessel width was analysed using the RAVE tool^47^.

### Pericyte coverage in the Matrigel model

We analysed pericyte coverage of blood vessels in Matrigel plugs by whole-mount staining for the pericyte marker NG2 and the EC marker CD105, and by measuring the NG2^+^ area as a percentage of the total CD105^+^ vessel area.

### Blood vessel tortuosity in the Matrigel model

Blood vessel tortuosity was scored semi-quantitatively by dividing × 20 images, taken on a Zeiss LSM 780 (Axio Observer.Z1) confocal microscope (Carl Zeiss), in four equal quarters. The CD105-stained vessel segments were then categorized into four distinct classes: (i) highly tortuous; (ii) mildly tortuous; (iii) nearly straight; and (iv) completely straight. A score was attributed to each image quarter and averaged per Matrigel plug. The scoring was done in a blinded manner.

### MSM-ATP development

The MSM of angiogenesis was used to study cellular dynamics during angiogenesis. In the MSM, ECs are modelled as ECagents, which consist of smaller memAgents that are connected by springs following Hooke's law, representing the actin cortex beneath the cell membrane^22^,^24^. These memAgents allow localized responses to the environment such as the extension or retraction of filopodia. The latest version of the model is extended with the Cellular Potts Model^27^, allowing differential adhesion-based dynamics to occur^9^. The model is well calibrated to *in vitro* and *in vivo* data and is well validated to simulate VEGF/Dll4/Notch signalling, cell shape changes, filopodia, junctional movements and EC rearrangement (for full details and methods see refs 9, 22, 24 and Supplementary Note). To enable simulation of ATP-driven processes, the MSM was extended with the modification that ATP levels govern actin and junctional dynamics (called MSM-ATP) by modulating three different cellular effectors alone or in combination. For each simulated mechanism the MSM-ATP effector(s) was (were) changed with a constant *k*.

E^FIL^: varying the probability of filopodia extension


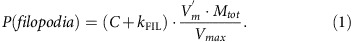


where: 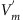 represents the memAgent's active VEGFR2, *M*_tot_ represents the current total number of memAgents of the ECagent, *V*_max_ represents an ECagent's maximal VEGFR2 capacity, *C* represents the strength of the VEGFR2-actin activation signal

E^COR^: varying the probability of junctional cortex protrusion formation


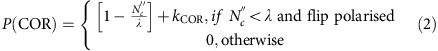


where: 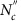 represents the ECagent's effective active Notch level, λ controls cellular migratory capacity

E^ADH^: changing the cellular adhesion levels


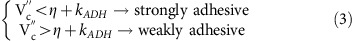


where: 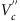 represents the ECagent's effective active VEGFR2 level, *η* represents a calibrated threshold to classify cells as strongly or weakly adhesive

### Quantifying tip cell competition

To compare the *in silico* simulations with the *in vitro* EC spheroid assay results, we developed an analogical evaluation method termed the ‘Artificial Brian' analysis method. In the EC spheroid assay, sprouting is stopped after 24 h by fixing the cells with paraformaldehyde (Carl Roth, Karlsruhe, Germany). Next, phase contrast microscopy is used to check the shape of the tip cell and whether it is attached to the sprout. Red and green fluorescence microscopy is used to determine which cell is at the tip position. The latter is determined by the relative position of cell bodies to each other rather than taking into account cytoskeletal protrusions such as filopodia. Accordingly, for the *in silico* simulations, we developed an analytical method to identify which cell is spearheading the *in silico* sprout as tip cell. We assigned 10 grid sites, which can be occupied by memAgents, to the front of the sprout, and then quantified how many of the 10 grid sites were occupied by memAgents for each cell. The cell with the most memAgents occupying the 10 grid sites was considered to be the tip cell, as this cell moved its cell body the most to the front. In case these 10 front grid sites were evenly occupied by different cells, the cell with the highest VEGFR2 activity was considered to be the tip cell. Next, we thoroughly tested if there is a difference in the relative contribution to the tip when the simulations are assessed at 24 h (2,880 timesteps as 1 timestep=30 s) or after the typical 125 h (15,000 timesteps) analysis time point used in ref. 9. No difference was observed and we therefore used the 125 h analysis time point as longer simulation runs would yield more information regarding sprouting/overtaking behaviour.

### Scoring of EC adhesive strength

At each simulation timestep, the levels of active VEGFR2 (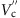 parameter^22^), which represent the levels of phosphorylated VEGFR2, are evaluated. These levels are then compared with a threshold in order to classify cells as strongly or weakly adhesive in the MSM-ATP in a binary fashion (*η* parameter^9^; 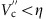: strongly adhesive, 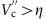: weakly adhesive). The adhesive strength as well as the genotype (isWT versus isPFKFB3^KD^) of each cell is tracked and saved to an output file, allowing us to link the adhesive strength to a cell's properties and to compare adhesion properties between sprouts in different conditions.

### *In silico* migration

At each simulation timestep, an *in silico* cell's centre of mass (COM) is calculated and stored in the x, y and z direction by averaging their memAgents' position in the respective directions. The COM in the *x*-direction (COM_x; low and high COM_x values, respectively, represent the rear and front of the sprout) is used to evaluate the distance covered by a cell. The absolute difference in a cell's COM_x values between two subsequent timesteps is stored and added to the total migrated distance of that cell. As such, we track the distance each cell covers during a simulation, allowing us to compare the migratory properties of different cells (for example, isWT vs isPFKFB3^KD^) in similar/different conditions (for example, VEGF levels).

### S&P pattern scoring

At each timestep after the onset of cellular rearrangements in the MSM-ATP (an initial stabilization period of 1,000 timesteps during which no cell movement is allowed, to permit the formation of a stable S&P pattern of active and inhibited cells^9^), all ECagents are tested for three properties: their active levels of Dll4 and VEGFR2 and the status of their actin pool. First, the Dll4 and VEGFR2 signalling activities of each ECagent are assessed to evaluate whether they meet the respective thresholds to be classified as an activated cell. If so, its actin pool will subsequently be assessed. If the actin threshold for activated cells is met, the number of ‘sprout active cells' is increased. However, to decrease the stringency of the S&P pattern scoring (too much information about the stabilization and maintenance of a S&P pattern would otherwise be lost), we introduce the term ‘stunted active cell'. This is a cell that meets the Dll4- and VEGFR2-signalling activity thresholds but not the actin threshold. Also the number of ‘stunted active cells' is evaluated. If for the whole sprout, the number of ‘active cells' (the sum of ‘stunted active cells' and ‘sprout active cells') equals 3 (that is, the number of ‘active cells' in an ideal S&P pattern in the used simulation set-up of 10 ECagents), a stable S&P pattern is possibly obtained/maintained. Whether a S&P pattern is classified as stable depends on the final test, which checks whether ‘active cells' are adjacent to each other. If the latter is true, the parameter recording the stabilizing time is incremented with one timestep, as such tracking the intermittent time between two subsequent S&P patterns. If not, a stable S&P pattern is obtained/maintained and the time during which it is maintained is stored. If the S&P pattern is newly generated, the time it took to stabilize into this pattern is used to calculate the average stabilizing time of S&P patterns and the parameter used to track the stabilizing time is reset to 0. Whenever the stable S&P pattern is lost, the time it lasted is used to calculate the average S&P pattern time. Throughout the whole simulation, all S&P pattern times are stored to eventually acquire the maximal time a S&P pattern could be maintained.

### Statistics

The data represent mean±s.e.m. of pooled experiments unless otherwise stated. The tip cell competition graphs depict representative *in vitro* or *in silico* experiments. Each *in vitro* experiment was performed at least 3 times and for all *in silico* experiments at least 50 simulations were run (unless otherwise specified). Each of these 50 simulations used a different random seed, which determines the initial positioning of the cells (for example, WT and isPFKFB3^KD^ cells) in the sprout. Statistical significance for tip cell competition graphs was calculated by performing Fisher's exact test (contingency test; mutant (green) or WT (red) present at tip). For all other graphs, statistical significance was calculated by standard *t*-test unless otherwise specified. All statistical analyses were performed in Graphpad Prism v6.0f. *P*<0.05 was considered significant. Throughout the manuscript the following symbols for statistical significance are used: *P<0.05, **P<0.01; ***P<0.001.

### Data availability

The authors declare that the data supporting the findings of this study are available within the article and its supplementary information files, or from the corresponding authors upon request. The executable MSM-ATP software as well as the patching algorithm are available on request.

## Additional information

**How to cite this article:** Cruys, B. *et al*. Glycolytic regulation of cell rearrangement in angiogenesis. *Nat. Commun.* 7:12240 doi: 10.1038/ncomms12240 (2016).

## Supplementary Material

Supplementary Information Supplementary Figures 1-7, Supplementary Note and Supplementary References

Supplementary Movie 1 MOVIE OF A SPROUT CONSISTING OF ISWT CELLS. Dynamic EC rearrangement (e.g. the initial tip cell (light green) is replaced by the light blue cell), is shown for an in silico sprout consisting of ten isWT cells (with two cells per crosssection). Each colour represents a different cell.

Supplementary Movie 2 MOVIE OF A 1:1 ISPFKFB3KD-FIL:ISWT SPROUT. isPFKFB3KD-FIL cells, which form fewer and shorter filopodia, and isWT cells are respectively shown in green and red.

Supplementary Movie 3 MOVIE OF A 1:1 ISPFKFB3KD-COR:ISWT SPROUT. isPFKFB3KD-COR and isWT cells are respectively shown in green and red. Both cell types present similar filopodia formation.

Supplementary Movie 4 MOVIE OF A 1:1 ISPFKFB3KD-ADH:ISWT SPROUT. The isPFKFB3KD-ADH and isWT cells are respectively shown in green and red. Both cell types present similar filopodia formation.

Supplementary Movie 5 MOVIE OF A 1:1 ISPFKFB3KD-FIL/COR:ISWT SPROUT. isPFKFB3KD-FIL/COR cells, which form fewer and shorter filopodia, and isWT cells are respectively shown in green and red.

Supplementary Movie 6 MOVIE OF A 1:1 ISPFKFB3KD-FIL/ADH:ISWT SPROUT. isPFKFB3KD-FIL/ADH cells, which form fewer and shorter filopodia, and isWT cells are respectively shown in green and red.

Supplementary Movie 7 MOVIE OF A 1:1 ISPFKFB3KD-COR/ADH:ISWT SPROUT. The isPFKFB3KD-COR/ADH and isWT cells are respectively shown in green and red. Both cell types display similar filopodia formation.

Supplementary Movie 8 MOVIE OF A 1:1 ISPFKFB3KD-ALL:ISWT SPROUT. The isPFKFB3KD-ALL cells, which form fewer and shorter filopodia, and isWT cells are respectively shown in green and red.

Supplementary Movie 9 EC SIGNALLING DYNAMICS IN AN ISWT SPROUT IN NORMAL VEGF LEVELS. The DLL4 expression levels, ranging from purple (low) to green (high), are shown for a sprout consisting of ten isWT cells exposed to normal VEGF levels. The ongoing signalling dynamics result in the formation of "salt and pepper" patterns of inhibited (purple) and activated cells (green).

Supplementary Movie 10 EC SIGNALLING OSCILLATIONS IN AN ISWT SPROUT IN 10- FOLD INCREASED VEGF LEVELS. The DLL4 expression levels, ranging from purple (low) to green (high), are shown for a sprout consisting of ten isWT cells exposed to 10-fold increased VEGF levels. The latter induce signalling oscillations during which all cells are simultaneously inhibited (purple) or activated (green) and prevent the formation of salt and pepper patterns of inhibited and activated cells.

Supplementary Movie 11 LIMITED EC REARRANGEMENT IN AN ISWT SPROUT IN 10- FOLD INCREASED VEGF LEVELS. EC rearrangement is limited (compare with Supplementary Movie 1) when a sprout consisting of ten isWT cells is exposed to 10-fold increased VEGF levels. For example, the purple cell remains at the same position throughout the movie. Each colour represents a different cell.

Supplementary Movie 12 NORMALIZED EC REARRANGEMENT IN AN ISWT SPROUT TREATED WITH A PFKFB3- AND VEGFR2-BLOCKER IN 10-FOLD INCREASED VEGF LEVELS. EC rearrangement is restored (compare with Supplementary Movie 11) when a sprout consisting of ten isWT cells is treated with a PFKFB3- and VEGFR2-blocker in conditions of 10-fold increased VEGF levels. For example, the light blue cell gets to the front of the sprout but subsequently becomes overtaken by trailing cells. Each colour represents a different cell.

Supplementary Movie 13 NORMALIZED EC SIGNALLING DYNAMICS IN AN ISWT SPROUT TREATED WITH A PFKFB3- AND VEGFR2-BLOCKER IN 10-FOLD INCREASED VEGF LEVELS. The ability to form "salt and pepper" patterns of inhibited (purple) and activated (green) cells of an isWT sprout in 10-fold increased VEGF levels is restored upon treatment with a PFKFB3- and VEGFR2-blocker (compare with Supplementary Movie 10). The colours represent the DLL4 expression levels, which range from purple (low) to green (high).
