# Supplementary material for: Glycolytic regulation of cell rearrangement in angiogenesis
Source: Nat Commun. 2016 Jul 20;7:12240. doi: 10.1038/ncomms12240 (PMC4961802; doi:10.1038/ncomms12240)
Supplement: Supplementary Information — Supplementary Figures 1-7, Supplementary Note and Supplementary References [file ncomms12240-s1.pdf]

SUPPLEMENTARY INFORMATION

SUPPLEMENTARY FIGURES

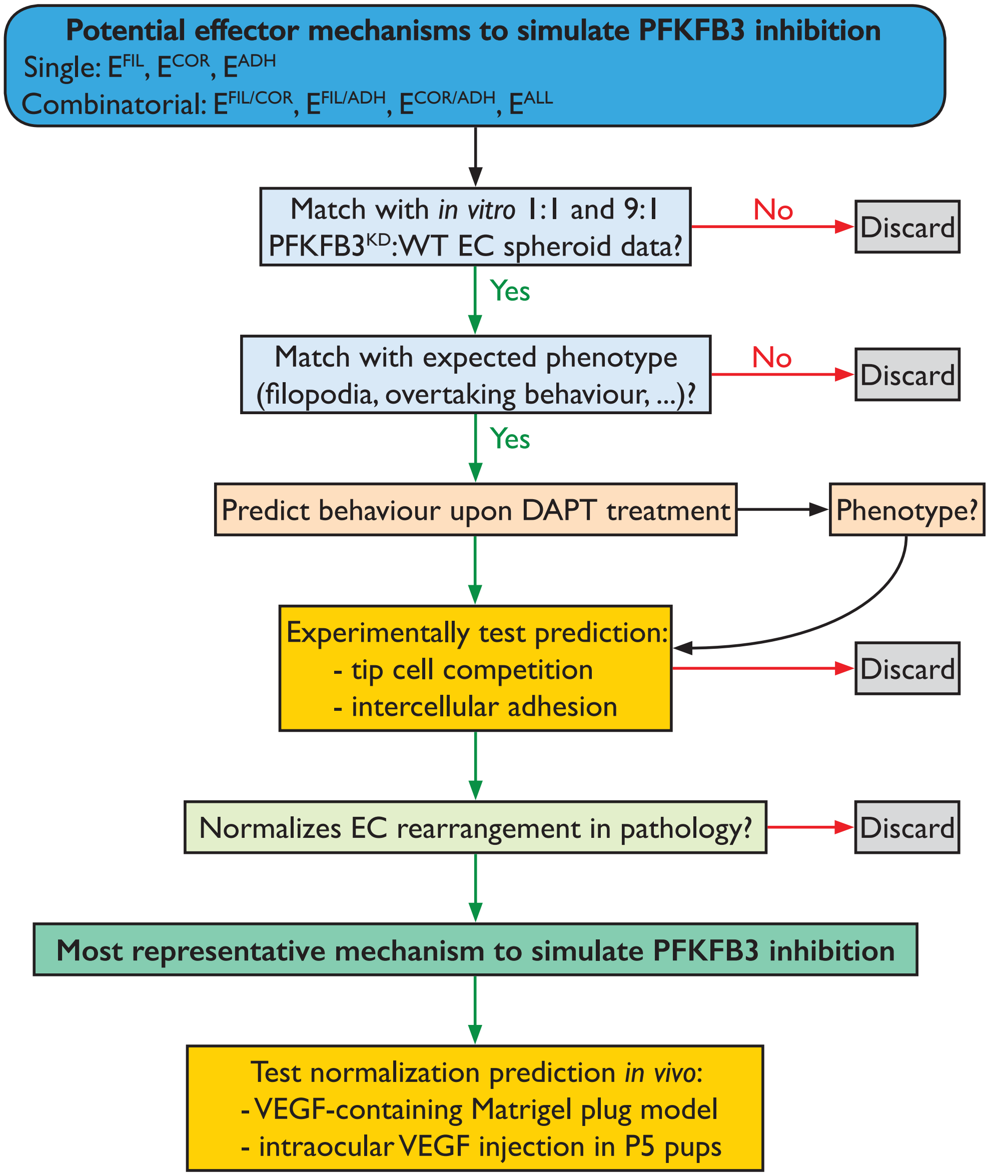

SUPPLEMENTARY FIGURE 1: DECISION TREE SHOWING THE STUDY PROCEDURE

The possible ATP-dependent effector mechanisms to simulate *in silico* PFKFB3 inhibition were subjected to multiple test scenarios and discarded if they did not match the experimentally obtained *in vitro* and *in vivo* phenotype(s) (light blue boxes). The MSM-ATP was optimized through multiple rounds of experimental validation (comparison of EC

behaviour and phenotypic traits in the MSM-ATP with EC spheroid competition data and filopodia formation, while ensuring normal EC dynamics in the simulated PFKFB3 inhibition mechanisms). This optimized MSM-ATP was then used to predict the behaviour of PFKFB3-inhibited cells in previously untested conditions (DAPT treatment; beige box) which were then experimentally verified (orange box) and to predict whether modifying those residual effector mechanisms that were selected to explain best the PFKFB3 inhibition phenotype, could normalize the perturbed EC dynamics in pathologically high VEGF conditions (green box). This ultimately led to one effector mechanism that is the most representative for the PFKFB3 inhibition phenotype, allowing us to acquire insights in the role of PFKFB3-driven glycolytic ATP production in EC rearrangement. Finally, we confirmed that blockade of PFKFB3, alone or together with VEGFR2 inhibition, normalizes vessel sprouting *in vivo*.

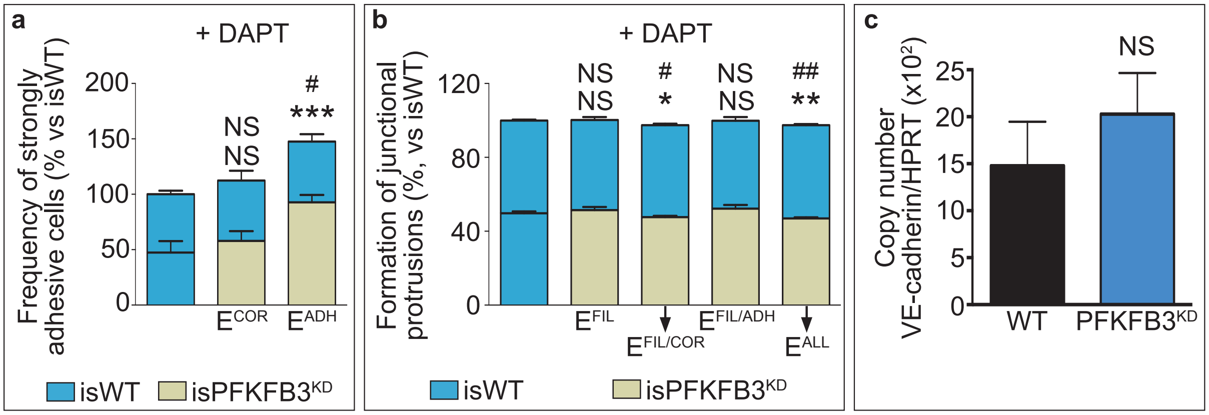

Supplementary Figure 2: Adhesion and cortical protrusions in DAPT and VE-cadherin expression

a. Analysis of EC adhesive strength of isWT cells in an isWT:isWT sprout (first bar) and of isPFKFB3^{KD-COR} and isPFKFB3^{KD-ADH} cells in a 1:1 isPFKFB3^{KD}:isWT mosaic sprout treated with DAPT. Varying E^{ADH}, but not E^{COR}, increased the fraction of isPFKFB3^{KD} cells that were classified as strongly adhesive, less motile cells (resulting in more heterogeneity in adhesion between sprout cells). Data are mean ± SEM; n=10; NS: not significant, ***p<0.001 *versus* total number of strongly adhesive cells in an isWT:isWT sprout; NS: not significant, #p<0.001: frequency of strongly adhesive isPFKFB3^{KD} mutants *versus* the expected frequency (50%); Student's *t*-test. **b.** Formation of cortical protrusions by isWT cells in a isWT:isWT sprout (first bar) and by isPFKFB3^{KD-FIL}, isPFKFB3$^{KD-FIL/COR}$, isPFKFB3$^{KD-FIL/ADH}$ and isPFKFB3^{KD-ALL} cells in a 1:1 isPFKFB3^{KD}:isWT mosaic sprout treated with DAPT. Data are mean ± SEM; n=4; NS: not significant, *p<0.05, **p<0.01 *versus* total number of cortical protrusions in an isWT:isWT sprout; NS: not significant, #p<0.05, ##p<0.01: relative number of cortical protrusions of isPFKFB3^{KD} mutants *versus* the expected frequency in isWT cells (50%); Student's *t*-test. **c.** VE-cadherin mRNA expression levels for control and PFKFB3^{KD} ECs. Data are mean ± SEM; n=3; NS: not significant; Student's *t*-test.

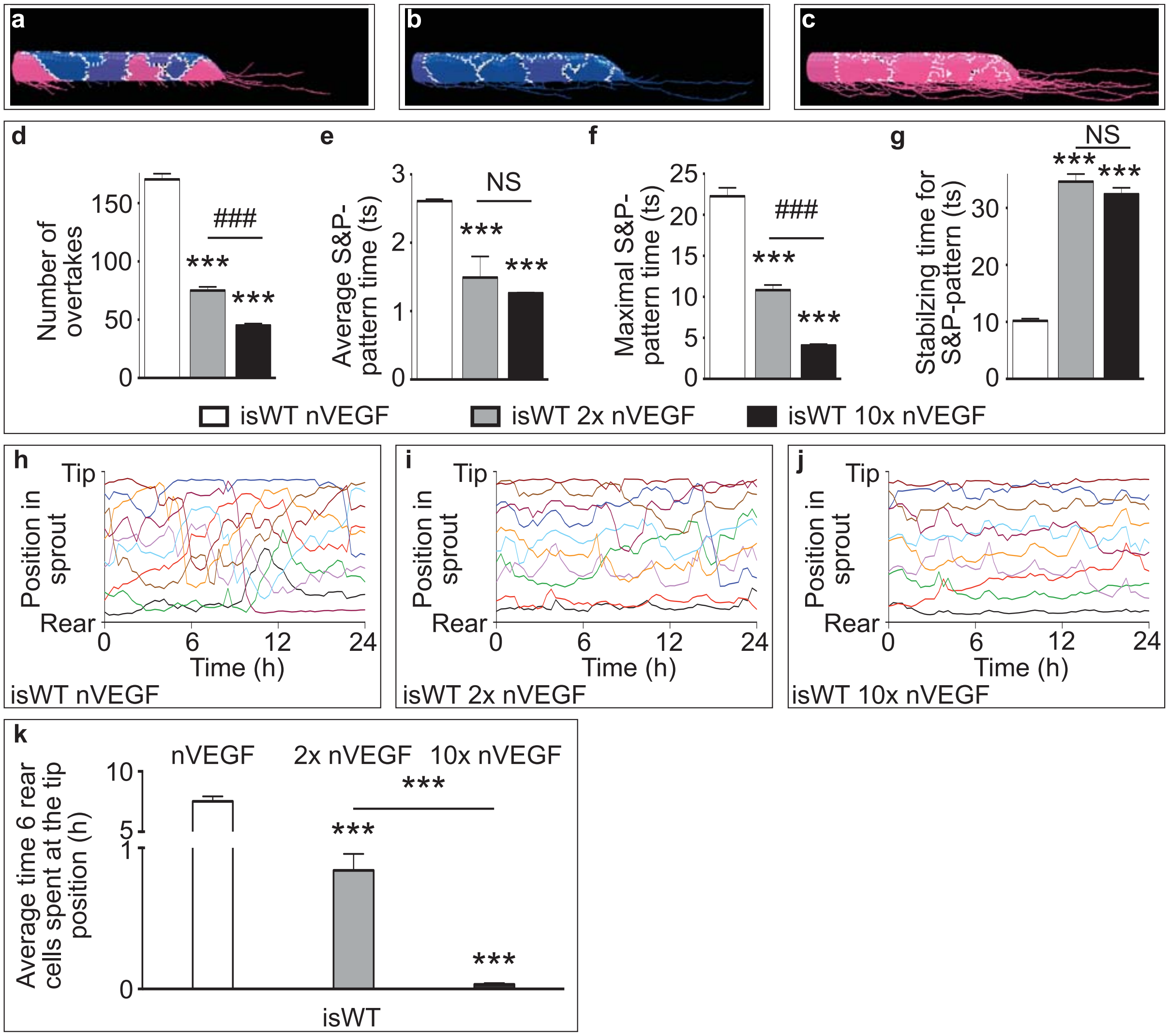

SUPPLEMENTARY FIGURE 3: THE BEHAVIOUR OF ISWT SPROUTS IN PATHOLOGICAL CONDITIONS

a. An ideal salt and pepper (S&P) pattern containing three active ECs (pink) surrounded by inhibited (blue) ECs. **b,c.** High VEGF levels induce signalling oscillations leading to synchronously inhibited (b) and activated (c) ECs. **d-g.** Number of overtakes (d), the average (e) and maximal time (f) during which a salt and pepper (S&P) pattern is maintained, and the time required to acquire a stable S&P pattern (g) for *in silico* WT (isWT) ECs, exposed to normal VEGF (nVEGF) levels (white) or VEGF levels that were elevated by 2- or 10-fold (2x nVEGF (grey) and 10x nVEGF (black) respectively) in non-mosaic sprouts. ts: timestep. Data are mean ± SEM; n=50; ***p<0.001 *versus* isWT in

nVEGF; NS: not significant, $^{###}p<0.001$ between 2x and 10x nVEGF; Student's *t*-test. **h-j.** Kymograph plots showing the movement of the ECs in isWT sprouts in different VEGF levels in function of the simulation time. Panels h, i and j represent an isWT sprout in nVEGF, 2x or 10x nVEGF levels, respectively. Each coloured line represents a different EC, the y-position denotes the relative position of the cell in the sprout. The higher the VEGF level, the fewer overtakes occur, observable as intersections between lines. **k.** The likelihood that the 6 cells, initially positioned at the sprout's rear end, reach and (temporarily) remain at the front of the sprout is dependent on the VEGF levels (white, grey and black bars denote normal (nVEGF), 2x and 10x nVEGF levels, respectively). Plotted is the summed average time these 6 cells spend at the tip of an *in silico* sprout of 10 isWT cells. Data are mean ± SEM; n=50; ***p<0.001; Student's *t*-test.

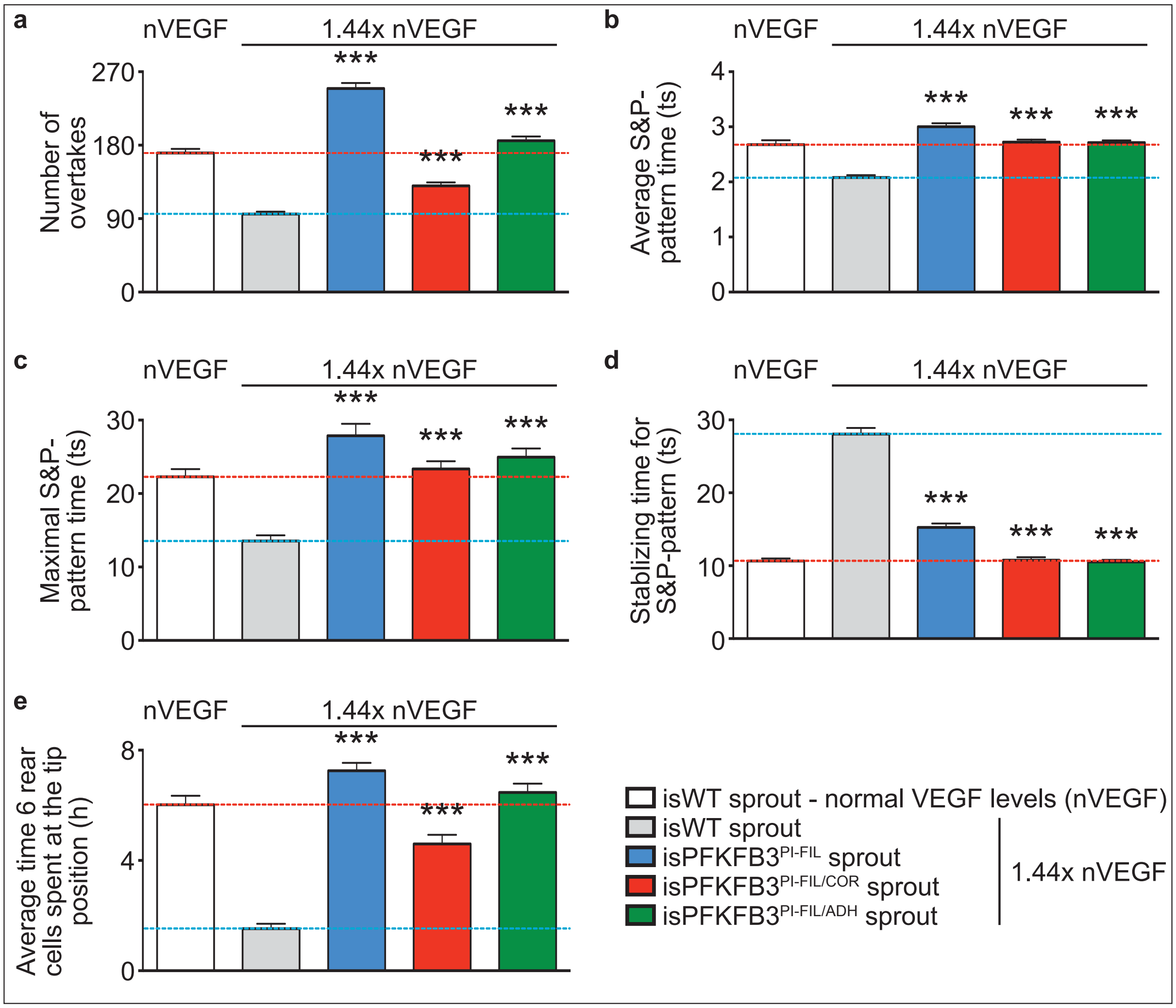

SUPPLEMENTARY FIGURE 4: EC DYNAMICS ARE COMPLETELY NORMALIZED IN 1.44-FOLD ELEVATED VEGF LEVELS

a-d. Number of overtakes (a), the average (b) and maximal time (c) during which a salt and pepper (S&P) pattern is maintained, and the time required to acquire a stable S&P-pattern (d) for *in silico* WT (isWT) ECs in normal VEGF (nVEGF) (white) or 1.44-fold increased VEGF (1.44x nVEGF - light grey) levels, and for ECs in which PFKFB3 was pharmacologically blocked (isPFKFB3^{PI}) simulated by modifying E^{FIL} (blue), $E^{FIL/COR}$ (red) and $E^{FIL/ADH}$ (green) in 1.44x nVEGF. The horizontal red and blue dotted lines show the particular values of an isWT sprout in normal and 1.44x VEGF levels respectively. ts: timestep. Data are mean ± SEM; n=50; ***p<0.001 *versus* isWT in 1.44x nVEGF;

Student's *t*-test. **e.** Likelihood that the 6 cells, initially positioned at the sprout's rear end, reach and (temporarily) remain at the front of a sprout of 10 *in silico* pharmacologically blocked PFKFB3 (isPFKFB3^{PI}) cells, modelled by varying E^{FIL} (blue), $E^{FIL/COR}$ (red) or $E^{FIL/ADH}$ (green) in 1.44-fold elevated VEGF (1.44x nVEGF) levels. Similarly, the white and light grey bar represent a simulated isWT sprout in normal VEGF (nVEGF) and 1.44x nVEGF levels, respectively. Plotted is the summed average time these 6 rear cells spend at the tip. The horizontal red and blue dotted lines show the particular values of an isWT sprout in normal and 1.44x VEGF levels respectively. Data are mean ± SEM; n=50; ***$p<0.001$ *versus* isWT in 1.44x nVEGF; Student's *t*-test.

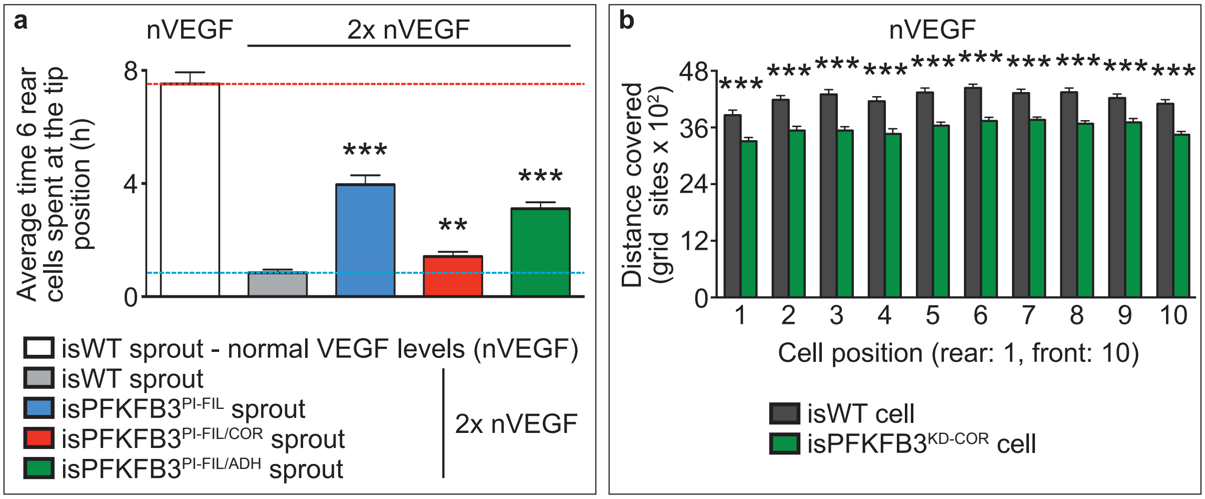

SUPPLEMENTARY FIGURE 5: EC SHUFFLING IN 2X VEGF LEVELS AND ISPFKFB3$^{KD\text{-}COR}$ CELL MIGRATION

a. Likelihood that the 6 cells, initially positioned at the sprout's rear end, reach and (temporarily) remain at the front of a sprout of 10 *in silico* pharmacologically blocked PFKFB3 (isPFKFB3^{PI}) cells, modelled by varying E^{FIL} (blue), $E^{FIL/COR}$ (red) or $E^{FIL/ADH}$ (green) in 2x VEGF levels (2x nVEGF). Similarly, the white and grey bars represent a simulated isWT sprout in normal VEGF (nVEGF) and 2x nVEGF levels, respectively. Plotted is the summed average time these 6 rear cells spend at the tip. The horizontal red and blue dotted lines show the particular values of an isWT sprout in normal and 2x VEGF levels respectively. Data are mean ± SEM; n=50; **p<0.01, ***p<0.001 *versus* isWT in 2x nVEGF (grey); Student's *t*-test. **b.** Analysis of EC migration in non-mosaic sprouts of isWT ECs or isPFKFB3$^{KD\text{-}COR}$ ECs in normal VEGF levels (nVEGF), showing that reducing cortical actin-based junctional protrusions (E^{COR}) results in reduced cell motility. Plotted is the distance a cell migrates, when this cell is positioned at the rear of the sprout (1) or progressively up to the front of the sprout (10) at the beginning of the simulation. Data are mean ± SEM; n=50; ***p<0.001; Student's *t*-test.

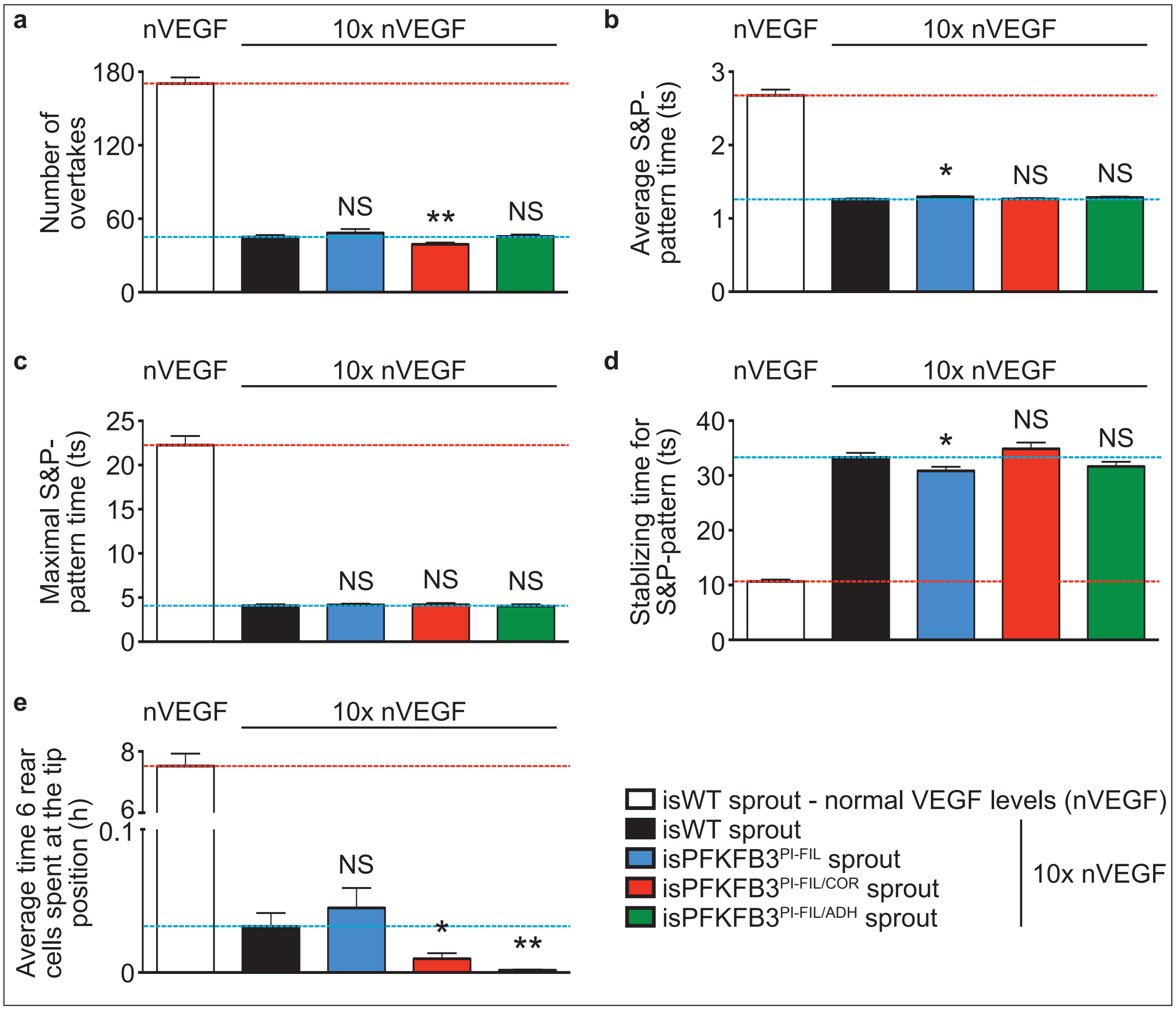

SUPPLEMENTARY FIGURE 6: THE EFFECTOR MECHANISMS DO NOT NORMALIZE EC DYNAMICS IN 10X VEGF LEVELS

a-d. Number of overtakes (a), the average (b) and maximal time (c) during which a salt and pepper (S&P) pattern is maintained, and the time required to acquire a stable S&P pattern (d) for *in silico* WT (isWT) ECs in normal VEGF (nVEGF) (white) or 10-fold increased VEGF (10x nVEGF - black) levels, and for ECs in which PFKFB3 was pharmacologically blocked (isPFKFB3^{PI}) simulated by modifying E^{FIL} (blue), $E^{FIL/COR}$ (red) and $E^{FIL/ADH}$ (green) in 10x nVEGF. The horizontal red and blue dotted lines show the particular values of an isWT sprout in normal and 10x VEGF levels respectively. ts: timestep. Data are mean ± SEM; n=50; NS: not significant, *p<0.05, **p<0.01 *versus* isWT

in nVEGF; Student’s *t*-test. **e.** Likelihood that the 6 cells, initially positioned at the sprout’s rear end, reach and (temporarily) remain at the front of a sprout of 10 *in silico* pharmacologically blocked PFKFB3 (isPFKFB3^{PI}) cells, modelled by varying E^{FIL} (blue), $E^{FIL/COR}$ (red) or $E^{FIL/ADH}$ (green) in 10-fold elevated VEGF (10x nVEGF) levels. Similarly, the white and black bars represent a simulated isWT sprout in normal VEGF (nVEGF) and 10x nVEGF levels, respectively. Plotted is the summed average time these 6 rear cells spend at the tip. The horizontal red and blue dotted lines show the particular values of an isWT sprout in normal and 10x VEGF levels respectively. Data are mean ± SEM; n=50; NS: not significant, *p<0.05, **p<0.01 *versus* isWT in 10x nVEGF; Student’s *t*-test.

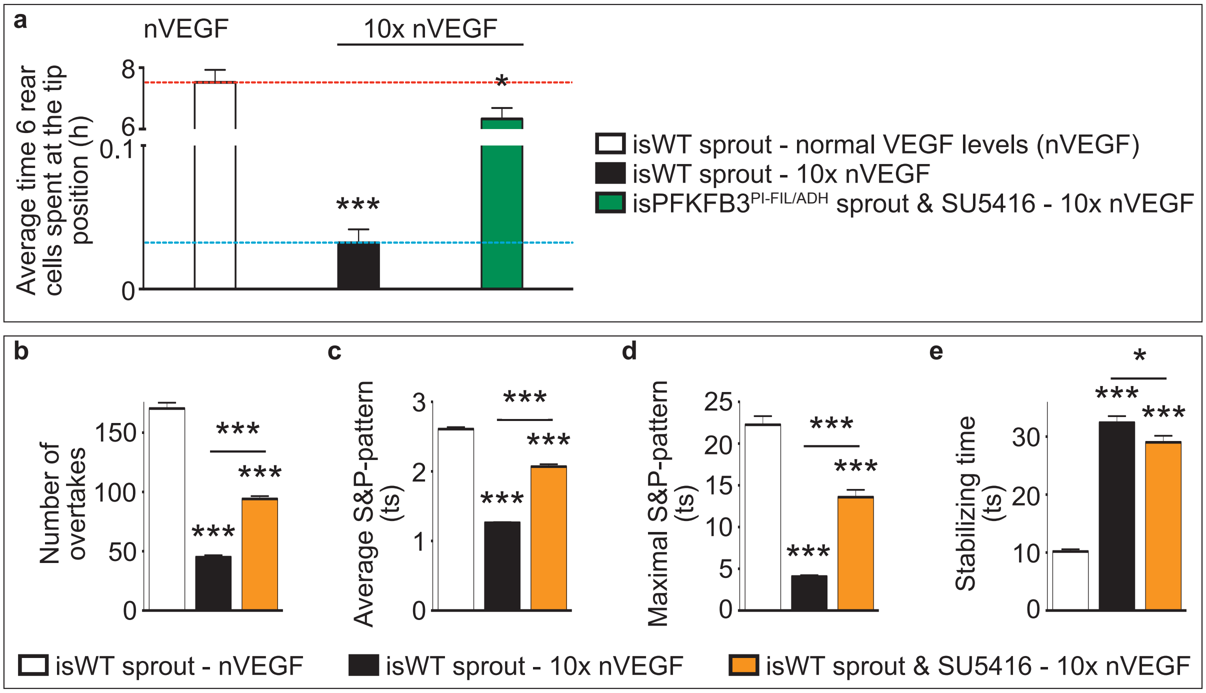

SUPPLEMENTARY FIGURE 7: ANTI-VEGFR2 TREATMENT ALONE AND IN COMBINATION WITH PFKFB3 BLOCKADE

a. Likelihood that the 6 cells, initially positioned at the sprout's rear end, reach and (temporarily) remain at the front of a sprout of 10 *in silico* pharmacologically blocked PFKFB3 (isPFKFB3^{PI}) cells, modelled by varying $E^{FIL/ADH}$, and treated with the tyrosine kinase inhibitor SU5416 (green) in 10-fold elevated VEGF (10x nVEGF) levels. Similarly, the white and black bars represent a simulated isWT sprout in normal VEGF (nVEGF) and 10x nVEGF levels, respectively. Plotted is the summed average time these 6 rear cells spend at the tip. The horizontal red and blue dotted lines show the particular values of an isWT sprout in normal and 10x VEGF levels respectively. Data are mean ± SEM; n=50; *p<0.05, ***p<0.001 *versus* isWT in nVEGF; Student's *t*-test. **b-e.** Number of overtakes (b), the average (c) and maximal time (d) during which a salt and pepper (S&P) pattern is maintained, and the time required to acquire a stable S&P pattern (e) for *in silico* WT (isWT) ECs in normal VEGF (nVEGF) (white) or 10-fold increased VEGF (10x nVEGF - black) levels, and for ECs treated with the tyrosine kinase inhibitor SU5416 in 10x nVEGF (orange). ts: timestep. Data are mean ± SEM; n=50; ***p<0.001 *versus* isWT in nVEGF, the statistical significance between the black and orange bars is also indicated (*p<0.05,

***$p<0.001$); Student's *t*-test.

GENERAL BACKGROUND OF THE MSM

The memAgent-Spring computational model of angiogenesis (MSM) is a hierarchical, cell-centred, agent-based model. It was initially developed to study tip cell selection and cell migration[1, 2, 3]. Later, it was extended with the Cellular Potts Model[4] (yielding the hybrid MSM-CPM) to investigate dynamic endothelial cell (EC) rearrangements in a vessel sprout[5]. The *in silico* vessel sprout consists of EC-agents (ECagents), which in turn are composed of membrane-Agents (memAgents). The latter represent sections of the cell membrane and are connected by springs that follow Hooke's law. These springs model the tension in the actin cortex underneath the cell membrane. The memAgents contain unique levels of the DLL4 ligand and the VEGFR2 and Notch1 receptors. The signalling pathways included in the MSM are implemented as simple, local rules, which allow the memAgents to autonomously interact with and respond to their local environment (the memAgent's Moore neighbourhood). Each ECagent knows at any time the position and protein expression levels of its memAgents and redistributes the receptors and / or ligands across its whole cell membrane at each timestep of the simulation. Hence, the localized memAgent responses determine the ECagent's features and yield emergent behavior at the level of the vessel sprout. The latter is the level that is used for assessing simulation results and predictions.

The signalling pathways of the MSM converge at VEGFR2. Tip cell selection depends on DLL4-Notch lateral inhibition and is modeled as a negative feedback loop, since active VEGFR2 signalling results in the upregulation of DLL4, which induces Notch signalling and subsequent VEGFR2 repression in adjacent cells. In contrast, cell migration is implemented as a positive feedback loop, since VEGFR2 signalling activity induces actin polymerization through the p38 and PI3K kinases and subsequent formation of filopodia (Equation 1). The latter are key for filopodia-led migration, which brings the ECagent closer to the VEGF source, which is modelled underneath the sprout as a linear gradient increasing towards the tip, and therefore increases VEGFR2 activity. Delays representing transcription / translation rates prevent the signalling effects (e.g. increased Dll4 expression) from being immediately effective in the cell. The resulting VEGFR2 and Notch signalling activities determine VE-cadherin-dependent adhesion levels (Equation 2, Mechanism 1 (M1)) and the ability to form polarized junctional cortex protrusions (Equation 3, Mechanism 2

(M2)). Heterogeneity in the latter two cell features has been shown to be the key driver of cell rearrangements in a sprout.

To simulate cell rearrangement with the MSM-CPM, at each simulation timestep, 8,000 copyflip attempts occur. During a copyflip, an ECagent locally protrudes its junctional cortex by moving a randomly chosen memAgent into a neighbouring ECagent's space. A simple set of rules determines whether such copyflip is accepted. Since cells strive to minimise the adhesive free energy, the probability of accepting a copyflip through M1 is higher if it reduces the system's free energy (Equation 4). The latter is determined by Equation 5 and depends on the junctional interfaces between cells as well as on the surface area of the cells. The energy cost of junctional interfaces is high, intermediate and low for junctional interfaces between weakly adhesive cells, strongly and weakly adhesive cells, and strongly adhesive cells, respectively (Equation 6). Hence, M1 tries to bring strongly adhesive cells together. When M1 rejects a copyflip, M2 can overrule this rejection if the copyflip is polarised towards the tip of the sprout and if the ECagent doing the copyflip has low Notch activity (Equation 3). In this way, M1 and M2 cooperate in pushing weakly adhesive cells forward and thus stimulate intercalation.

Overall, The MSM-CPM thus provides a well-suited testbed to study cell rearrangements and tip-stalk cell signalling in conditions of PFKFB3 inhibition. More information on parameter values can be found in [1, 2, 5].

$$P(filopodia) = C \cdot \frac{V'_m \cdot M_{tot}}{V_{max}} \tag{1}$$

where: C represents the levels of WASP/PIP2 activation
V'_m represents the memAgent's active VEGFR2
M_{tot} represents the present number of memAgents of the ECagent
V_{max} represents an ECagent's maximal VEGFR2 capacity

$$\begin{cases} \mathrm{V}_c'' < \eta \ \rightarrow strongly\ adhesive \\ \mathrm{V}_c'' > \eta \rightarrow \ weakly\ adhesive \end{cases} \tag{2}$$

where: V_c'' represents the ECagent's effective active VEGFR2 level
η represents a calibrated threshold to classify cells as strongly or weakly adhesive

$$P(pol.junc.cor.protrusion) = \begin{cases} 1 - \frac{N_c''}{\lambda} & , \quad \text{if } N_c'' < \lambda \text{ and flip polarised} \\ 0 & , \quad \text{otherwise} \end{cases} \tag{3}$$

where: N'' represents the ECagent's effective active Notch level
λ represents the level of difference of the mobility of cells

$$P(accept\ copyflip - M1) = \begin{cases} e^{\frac{-\Delta H}{T}} & , \quad if\ \Delta H > 0 \\ 1 & , \quad if\ \Delta H \ \leq 0 \end{cases} \tag{4}$$

where: ΔH represents the change in free energy
T represents the cell motility

$$H = \sum_{local} J(x,y) + \sum_{copier,flipper} \left(A_{cell} - A_{target}\right)^2 \tag{5}$$

where: H represents the free energy level
J represents the energy cost of the junctional interface
x represents ECagent x
y represents ECagent y
A_{cell} represents the current surface area of the ECagent
A_{target} represents the ideal, "target" surface area of an ECagent

$$J(x,y) = \begin{cases} 14, & if\ both\ ECagent\ x\ and\ y\ are\ weakly\ adhesive \\ 11, & if\ the\ adhesion\ level\ of\ ECagent\ x\ and\ y\ differ \\ 4, & if\ both\ ECagent\ x\ and\ y\ are\ strongly\ adhesive \end{cases} \tag{6}$$

where: $J(x,y)$ represents the energy cost of the junctional interface between ECagent x and ECagent y

GENERATION OF THE MSM-ATP

We modified three effectors of the MSM model in order to make them dependent on ATP levels by calibrating them to experimental tip cell competition data. Therefore, we varied them by introducing respectively the k_{FIL}-, k_{COR}-, and k_{ADH}-values as follows:

1) E^{FIL}: varying the probability of filopodia extension

$$P(filopodia) = (C + k_{FIL}) \cdot \frac{V'_m \cdot M_{tot}}{V_{max}} \tag{7}$$

where: V'_m represents the memAgent's active VEGFR2
M_{tot} represents the current total number of memAgents of the ECagent
V_{max} represents an ECagent's maximal VEGFR2 capacity
C represents the strength of the VEGFR2-actin activation signal and its wild type (WT) value equals 2.

2) E^{COR}: varying the probability of junctional cortex protrusion formation

$$P(COR) = \begin{cases} \left[1 - \frac{N''_c}{\lambda}\right] + k_{COR} & , \text{ if } N''_c < \lambda \text{ and flip polarised} \\ 0 & , \quad \text{otherwise} \end{cases} \tag{8}$$

where: N''_c represents the ECagent's effective active Notch level
λ controls cellular migratory capacity

3) E^{ADH}: changing the cellular adhesion levels

$$\begin{cases} \mathrm{V''_c} < \eta + k_{ADH} \rightarrow \textit{strongly adhesive} \\ \mathrm{V''_c} > \eta + k_{ADH} \rightarrow \textit{weakly adhesive} \end{cases} \tag{9}$$

where: V''_c represents the ECagent's effective active VEGFR2 level
η represents a calibrated threshold (the WT-value of η equals 200) to classify cells as strongly or weakly adhesive

We varied these *k*-values to modulate the physiological processes of filopodia extension, junctional cortex protrusions formation and intercellular adhesion in the model. This approach

allowed us to qualitatively assess the contribution of each single simple parameter to vessel sprouting, without a need to simulate every possible co-factor, kinase, phosphorylation site, etc involved in the physiological process. The latter would introduce too many unknown parameters, and our aim was to explore how the E^{FIL}, E^{COR} and E^{ADH} effectors (alone or together) regulated cell motion.

To simulate PFKFB3 silencing, we reduced the probability of filopodia extension by varying the k_{FIL}-value from 0 to -100%. Similarly, we reduced the probability of cortical junction formation by varying the k_{COR}-value from 0 to -50% (maximal effect). To simulate the reduced VE-cadherin endocytosis upon PFKFB3 silencing, we increased the threshold η from 2- to 25-fold (maximal effect) by using positive k_{ADH}-values. k_{FIL} and k_{COR} can be expressed in percentages as these modify probabilities, respectively of filopodia and junctional protrusion extension. k_{ADH} cannot be expressed in a unit as it modified a unit-less threshold used to classify cell as strongly or weakly adhesive in a binary fashion.

Simulating mosaic sprouts

EC sprouting in the *in vitro* spheroid EC competition assay is initiated by adding growth factors to EC spheroids. The EC spheroid contains a mix of ECs with different genotypes, each expressing a distinct fluorophore[6]. Similarly, for the simulations, we specify the ratio (e.g. 1:1 and 1:9) at the onset of the *in silico* simulation. The ECs are positioned at the beginning of the simulation at random positions in each of the 50 simulations ran per condition by making use of random seeds (of the Intel ICC compiler).

Simulating DAPT

DAPT is a γ-secretase inhibitor that blocks the release of the Notch intracellular domain and therefore inhibits Notch signalling. To simulate the DAPT treatment, we allow normal DLL4-Notch signalling to take place but set the active Notch levels (*N″*) to zero in all ECagents. Hence, regardless of the amount of DLL4 presented by adjacent ECagents, an ECagent will not have any Notch signalling activity and will thus not experience downstream effects on gene expression and

cell dynamics.

K-VALUES, CALIBRATED TO MATCH THE EXPERIMENTAL DATA OBTAINED WITH THE PFKFB3^{KD} CELLS, USING THE MSM-ATP

E^{FIL} (representing filopodial F-actin), E^{COR} (referring to cortical actin), and E^{ADH} (denoting intercellular adhesion) determine respectively the probability of filopodia extension, the formation of polarized junctional protrusions and cellular adhesion levels. E^{FIL} and E^{COR} are reduced, while E^{ADH} is increased by varying k_{FIL}, k_{COR} and k_{ADH}, respectively (see Equation 7-9). For the combinatorial mechanisms $E^{FIL/COR}$, $E^{FIL/ADH}$, $E^{COR/ADH}$ and E^{ALL}, multiple effectors are varied simultaneously. To calibrate these combinatorial mechanisms, we used the identified matching *k*-values of the single mechanisms as initial guiding values. We fixed one of the effector mechanisms contributing to the combinatorial mechanism by resetting its *k*-value to a value, lower than the *k*-value identified for the corresponding single mechanism (Table 1). We then varied the *k*-value(s) of the other contributing effector mechanism(s) by simulating values that are also lower than those identified for the single mechanism(s). We thus aimed to identify a combination of *k*-values that: (i) matched the competition results in both 1:1 and 9:1 isPFKFB3^{KD}:isWT chimeras, (ii) are sufficiently distinct (by at least 33%) from the respective *k*-value in the single isPFKFB3^{KD} mechanism, and (iii) are changed to a similar extent (thereby preventing that one effector would be more dominant in generating the phenotype than the other(s)). If no combination of *k*-values could be identified to satisfy these constraints, we changed the fixed *k*-value by a small amount and repeated this process until a set of *k*-values was found that fulfilled the abovementioned criteria. For each combinatorial mechanism, at least three of these iterations (i.e. changing the fixed *k*-value for at least three times) were required.

For example, for isPFKFB3$^{KD\text{-}FIL/COR}$, we selected a k_{COR}-value that reduces cortical protrusion formation less than the k_{COR}-value identified for the single isPFKFB3$^{KD\text{-}COR}$ mechanism, and then varied k_{FIL} across a range of values that reduce E^{FIL} less than the calibrated k_{FIL}-value does for the single isPFKFB3$^{KD\text{-}FIL}$ mechanism. In more detail, we first set k_{COR} to a value of -12% (for the single isPFKFB3$^{KD\text{-}COR}$ mechanism, k_{COR} is -19%) and simulated k_{FIL}-values between -7.5% and -4% (for the single isPFKFB3$^{KD\text{-}FIL}$ mechanism, k_{FIL} is -11.5%). However, we could not find a k_{FIL}-value in this

range that satisfied the constraints when k_{COR} = -12%. We therefore fixed k_{COR} to -10%. Again, no matching k_{COR}-k_{FIL} combination could be identified, so we reset k_{COR} to -8% and combined this value with a range of k_{FIL}-values. After four iterations, we found a matching combination, in which k_{COR} was -7% and k_{FIL} was -5%. We followed a similar procedure for the three other combinatorial mechanisms to identify the *k*-values shown in the table below. The listed *k*-values were identified to match the data on tip cell competition experimentally obtained with 1:1 and 9:1 mosaic PFKFB3^{KD}:WT sprouts (see Fig. 1c of the main article) and [6].

Table 1: Calibrated *k*-values used to simulate isPFKFB3^{KD}

Mechanism	k_{FIL} (%)	k_{COR} (%)	k_{ADH}
isPFKFB3$^{KD\text{-}FIL}$	-11.5	0	0
isPFKFB3$^{KD\text{-}COR}$	0	-19	0
isPFKFB3$^{KD\text{-}ADH}$	0	0	310
isPFKFB3$^{KD\text{-}FIL/COR}$	-5	-7	0
isPFKFB3$^{KD\text{-}FIL/ADH}$	-7.5	0	195
isPFKFB3$^{KD\text{-}COR/ADH}$	0	-8	130
isPFKFB3$^{KD\text{-}ALL}$	-4	-6	100

Calibration to simulate pharmacological PFKFB3 inhibition

To model the pharmacological PFKFB3 blockade (isPFKFB3^{PI}), we re-calibrated the remaining mechanisms (E^{FIL}, $E^{FIL/COR}$ and $E^{FIL/ADH}$) to the EC migration data upon treatment with a PFKFB3 blocker. Since the pharmacological PFKFB3 inhibitors 3PO and YN1 reduced *in vitro* EC migration more than PFKFB3 silencing (PFKFB3^{KD}) (by 48% *versus* 19%), we re-calibrated the isPFKFB3 mechanisms so that EC migration of isPFKFB3^{PI} cells matched the migration of ECs treated with 3PO and YN1. We therefore adapted the *k*-values of the isPFKFB3^{KD} cells so that the difference in migration between the isPFKFB3^{PI} and isPFKFB3^{KD} cells was 29% (i.e. the difference between 48 and 19%). The calibrated *k*-values are shown in the table below.

Table 2: Calibrated *k*-values used to simulate isPFKFB3[PI]

MECHANISM	κ_{FIL} (%)	κ_{COR} (%)	κ_{ADH}
isPFKFB3$^{PI\text{-}FIL}$	-37.5	0	0
isPFKFB3$^{PI\text{-}FIL/COR}$	-30	-20	0
isPFKFB3$^{PI\text{-}FIL/ADH}$	-35	0	250

SIMULATING THE VEGFR2 INHIBITOR SU5416

To model VEGFR2 inhibition by SU5416, we reduced the VEGFR2 signalling capacity by dividing $\mathbf{V_c''}$ by a constant value x. To obtain complete normalization of EC rearrangement and signalling dynamics upon combined *in silico* treatment with 3PO and SU5416, x was set to 6.

QUANTIFYING TIP CELL COMPETION *IN SILICO* (“ARTIFICIAL BRIAN”)

We provide here more details about our newly developed computational quantification method, which we based on the criterion that the leading cell of the sprout is the one whose cell body occupies most of the front of the sprout, without however considering cytoskeletal protrusions such as filopodia (identical to how the *in vitro* EC spheroid competition assay was manually quantified). In the MSM-ATP, a vessel sprout is modeled as a cylindrical mesh. While the rear of the sprout (left on the x-axis) is open-ended as if it is connected to the vascular plexus, the front of the sprout (right on the x-axis) is closed. Simulated ECs can change their position within the cylindrical mesh, but the mesh itself is fixed in space. Hence, the front of the sprout is always at the same location, indicated by x_{MAX}. MemAgents represent regions of the simulated EC membrane. The sprout front line at x_{MAX} contains 10 grid sites that can be occupied by memAgents (indicated by arrowheads in the frontal view, not visible in the zoomed side view; arrows denote filopodia; Figure 1). To assess which EC is spearheading the *in silico* sprout (and hence is the *bona fide* tip cell), we quantified which EC’s memAgents occupied most of the 10 front grid sites. As such, the EC with the most memAgents at the front has moved its cell body most to the front and will be counted as a “tip cell”. In the example (Figure 1), the green cell would be considered a tip cell, since there are 6 green and 4 blue memAgents (arrowheads) at the front of the sprout.

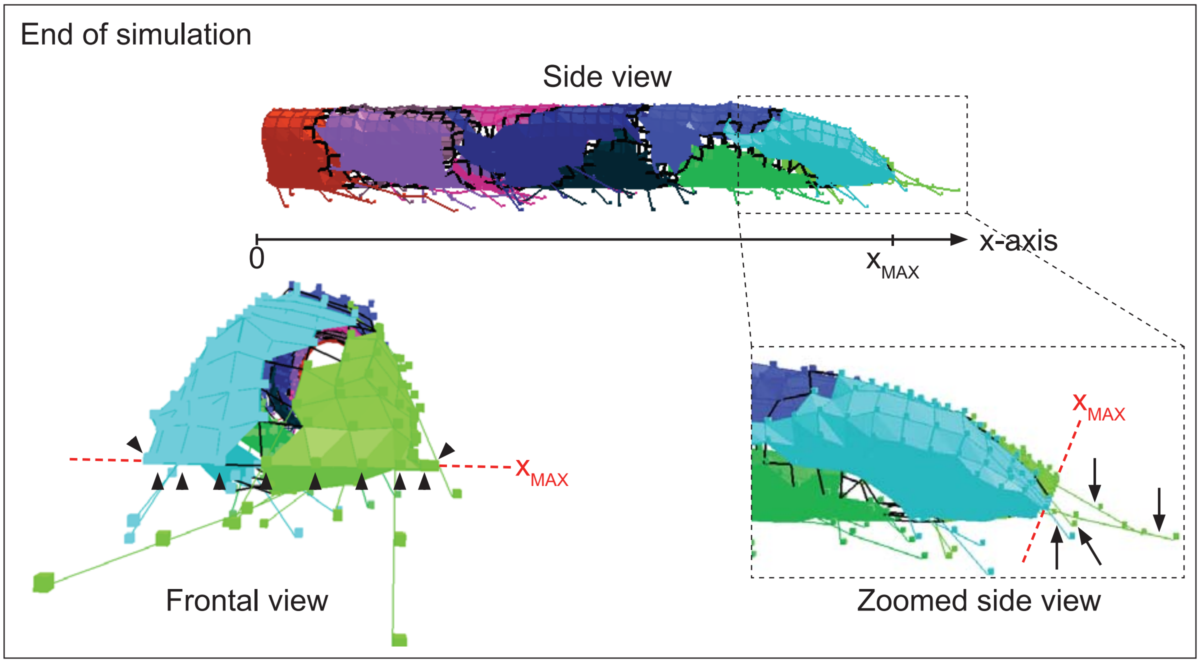

Figure 1: Computational tip cell quantification method ("Artificial Brian"). Arrowheads in the zoomed frontal view denote the 10 grid sites that can be occupied by memAgents at the front of the sprout (x_{MAX}); arrows in the zoomed side view denote filopodia.

References

1. Bentley K, Gerhardt H, Bates PA. Agent-based simulation of notch-mediated tip cell selection in angiogenic sprout initialisation. *J Theor Biol* **250**, 25-36 (2008).

2. Bentley K, Mariggi G, Gerhardt H, Bates PA. Tipping the balance: robustness of tip cell selection, migration and fusion in angiogenesis. *PLoS Comput Biol* **5**, e1000549 (2009).

3. Bentley K, Harrington K, Regan ER. Can active perception generate bistability? Heterogeneous collective dynamics and vascular patterning. *Proceedings of Artificial Life* **XIV**, 328-335 (2014).

4. Graner F, Glazier JA. Simulation of biological cell sorting using a two-dimensional extended Potts model. *Phys Rev Lett* **69**, 2013-2016 (1992).

5. Bentley K*, et al.* The role of differential VE-cadherin dynamics in cell rearrangement during angiogenesis. *Nat Cell Biol* **16**, 309-321 (2014).

6. De Bock K*, et al.* Role of PFKFB3-driven glycolysis in vessel sprouting. *Cell* **154**, 651-663 (2013).
